# Taxonomy of African driver ants (Hymenoptera, Formicidae, *Dorylus nigricans* species group) west of the Dahomey Gap

**DOI:** 10.3897/zookeys.1290.192534

**Published:** 2026-08-25

**Authors:** Caspar Schöning, Raphael Weniger, William Gotwald Jr., Lars Vilhelmsen, Jacobus J. Boomsma, Daniel J. C. Kronauer

**Affiliations:** 1 Section for Ecology and Evolution, Department of Biology, Universitetsparken 15, 2100 Copenhagen, Denmark Section for Ecology and Evolution, Department of Biology Copenhagen Denmark; 2 Senckenberg Museum of Natural History, Am Museum 1, D-02826 Görlitz, Germany Senckenberg Museum of Natural History Görlitz Germany; 3 TUD Dresden University of Technology, 01062 Dresden, Germany TUD Dresden University of Technology Dresden Germany; 4 Department of Biology, Utica College, 1600 Burrstone Road, Utica, NY 13502, USA Department of Biology, Utica College Utica United States of America; 5 Natural History Museum, SCIENCE, University of Copenhagen, Sølvgade 83, DK-1307 København K, Copenhagen, Denmark Natural History Museum, SCIENCE, University of Copenhagen Copenhagen Denmark; 6 Laboratory of Social Evolution and Behavior, The Rockefeller University, 1230 York Avenue, New York, NY 10065, USA Laboratory of Social Evolution and Behavior, The Rockefeller University New York United States of America; 7 Howard Hughes Medical Institute, 1230 York Avenue, New York, NY 10065, USA Howard Hughes Medical Institute New York United States of America

**Keywords:** Army ants, Dorylinae, Formicidae, male-worker-matching, morphometrics, West Africa

## Abstract

Driver ants (*Dorylus nigricans* species group) are keystone species in forest and savanna ecosystems across tropical Africa. However, their study has been hampered by considerable taxonomic confusion. A multitude of worker forms has been described under the name *Dorylus nigricans* Illiger, 1802, even though the link between the name-bearing type specimens, two males from Sierra Leone, and the respective conspecific workers has never been established. Extensive surveys of driver ants were first conducted at numerous West African sites from Senegal to Ghana. These yielded a total of only three species recognisable by morphological differences. We then examined the two *D. nigricans* syntypes and found that they belong to different species. By analysing mitochondrial and nuclear DNA sequences of males and workers from Taï National Park (Ivory Coast), males with a phenotype similar to that of the larger syntype specimen were matched to workers originally described under the name *Anomma* arcens by Westwood (1847). This larger syntype male is designated as the lectotype of *D. nigricans* Illiger, 1802, so that *Anomma arcens* Westwood, 1847 is synonymised under *D. nigricans*. A worker neotype for the species originally described as *Anomma burmeisteri* Shuckard, 1840, whose type specimen was lost, is designated. A key to the large workers of the three driver ant species occurring in this region, *D. burmeisteri*, *D. nigricans*, and *D. mayri*, is provided. By clarifying the taxonomy of the regional driver ant fauna, we resolve a centuries-old puzzle and pave the way for a comprehensive revision of the entire species group.

## Introduction

**Figure 1.**
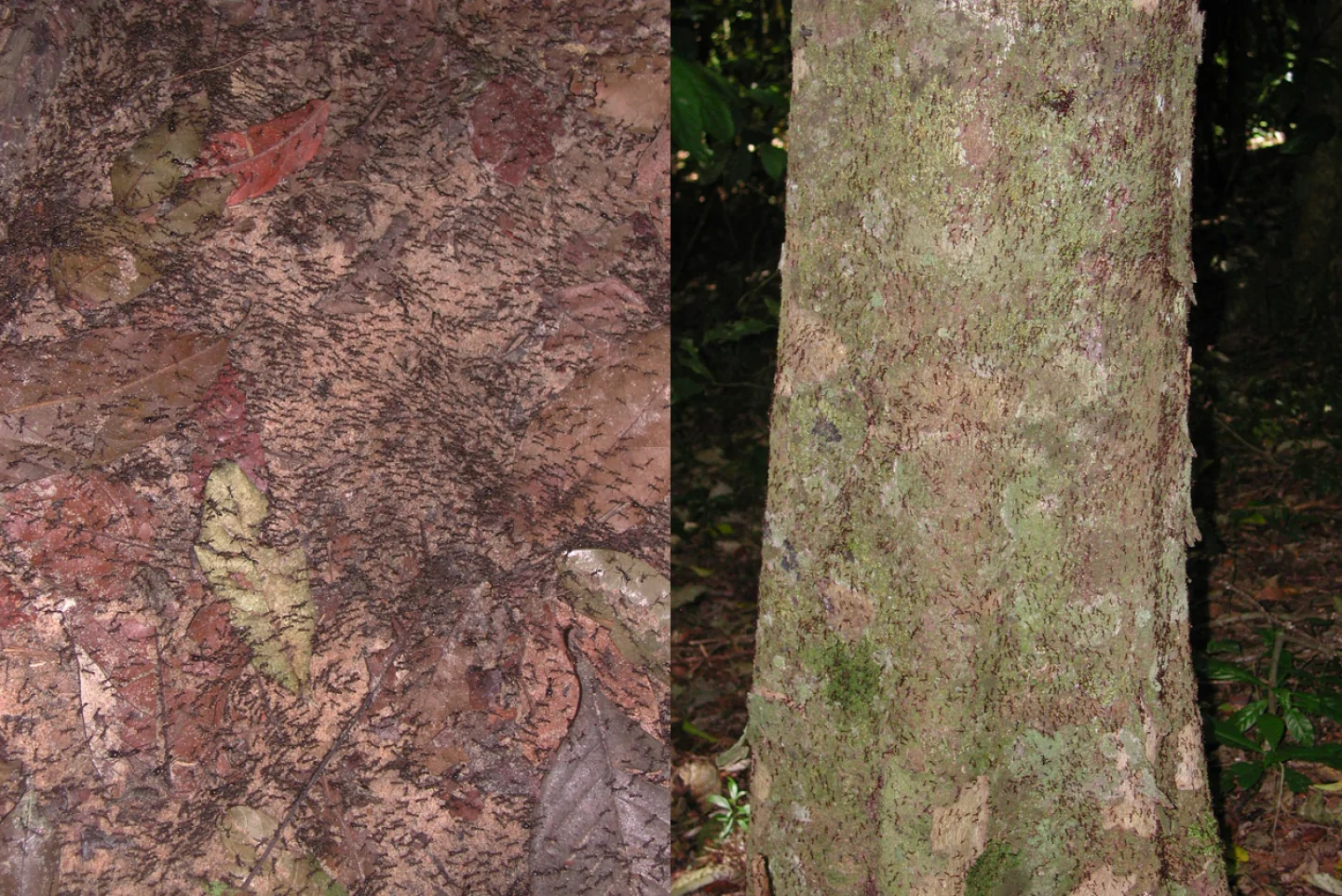
Swarm raiding, the typical foraging behaviour of driver ants. Left picture taken in Taï National Park, Ivory Coast; right picture taken at Mount Kenya, Kenya.

**Figure 2.**
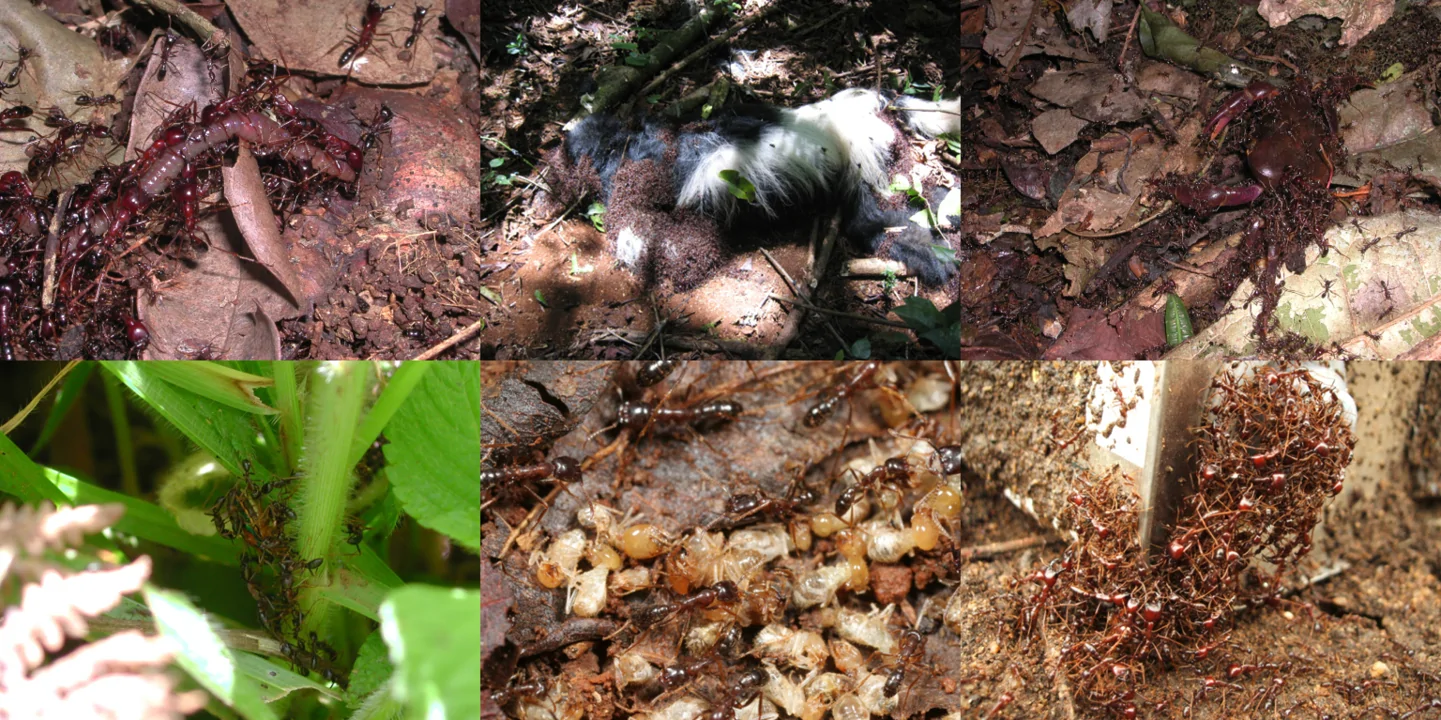
Examples of driver ant food types. Upper row from left to right: a *Polytoreutus* earthworm, the carcass of a black-and-white colobus monkey, a fresh-water crab; lower row from left to right: a true bug, *Odontotermes* termites, cooking oil.

Advancing in search of prey, a raid swarm of African driver ants (*Dorylus nigricans* species group) covers the forest floor like a black carpet. Such raid swarms can be 20 m or more wide (Leroux 1982). Raiding ants also climb the vegetation, attacking herbivorous insects, spiders, and other animals they encounter there. Together with the column connecting the swarm to the nest, the raid resembles the ‘pseudopodium of a giant amoeba’ (Hölldobler and Wilson 2009) retrieving prey from areas at a distance of up to 250 m (Raignier and Van Boven 1955). To anyone who has ever spent time watching this fascinating spectacle, it is immediately clear why Savage (1847) coined the term ‘driver ant’ for these fierce predators. Because any animal capable of movement tries to flee from the approaching ants, the raid swarm drives before it a large number of invertebrates (and to a lesser extent vertebrates) seeking to escape (Fig. 1). The masses of flushed invertebrates in turn attract both opportunistic and specialised birds feeding on them (Willis and Oniki 1978; Keith et al. 1992; Peters et al. 2008). Driver ants are distributed throughout forests and other humid habitats such as woodlands and agroforestry areas of tropical Africa (ca 15°N, 20°S). With a total body size of up to 6 cm (Raignier et al. 1974), driver ant queens are the largest extant ants and have an enormous egg-laying capacity (an estimated 3–4 million eggs per month in *Dorylus wilverthi*, Raignier and Van Boven 1955). Driver ant colonies may contain as many as 9 million adult ants and have a total fresh mass of up to 50 kg (Leroux 1982), which is equivalent to the body mass of a female leopard (*Panthera pardus*, Kingdon 1997). All driver ant species are carnivorous and have a diverse prey spectrum (Gotwald 1995; Schöning and Moffett 2007; Schöning et al. 2008a), harvesting solitary insects (predominantly immature life stages), earthworms, spiders, molluscs (snails and slugs), millipedes, centipedes, crustaceans (crabs, woodlice), occasionally large food items such as social insect colonies (ants, honey bees, termites, wasps), vertebrates (frogs, mice, fledgling birds, carcasses), and to a lesser degree food types of plant origin (Fig. 2).

The conspicuous swarm raids of driver ants may contribute to the maintenance of arthropod diversity in tropical forests by creating a mosaic of patches at different stages of recovery as has been proposed for the Neotropical species *Eciton burchellii* (Franks and Bossert 1983; Peters et al. 2013). Many parasitic or commensal invertebrate species (so-called myrmecophiles) are directly dependent on driver ants as hosts (Kistner 1982), and some vertebrates such as the specialised antbirds and parasitic snakes of the genus *Afrotyphlops* are closely associated with army ants as well (Gotwald 1995). On the other hand, chimpanzees, gorillas, honey badgers, mongooses, and pangolins prey on driver ants (Gotwald 1995; Kingdon 1997). Driver ants may also play important roles in soil turnover. At Mount Kenya, colonies bring on average almost 35 kg of soil to the surface during each temporary stay, and this soil is mixed with non-digestible chitinous prey remains discarded by the ants (Schöning et al. 2005a). Their roles as predators, prey, soil movers, and hosts of parasites and commensals strongly indicate that driver ants play key roles in Afrotropical forests (Gotwald 1995), but ecological studies of these fascinating ants have been hampered by considerable taxonomic confusion.

The driver ant species *Dorylus burmeisteri* Shuckard, 1840 was described based on a single worker collected in Sierra Leone, and was the basis for erecting a new genus, *Anomma*. Savage (1847, 1849) was the first to produce a detailed report of driver ant behaviour, and the specimens he collected close to Cape Palmas (Liberia) were described as *Anomma arcens*, Westwood 1847. *Anomma rubella* Savage, 1849 was subsequently described from what is today Gabon. This species showed the same hunting behaviour as *A. burmeisteri* and *A. arcens*, which established the common name ‘driver ant’ for all species foraging in massive swarm raids both on the forest floor and in the vegetation (Savage 1847, 1849). These species are often described as ‘epigaeic’ (Schöning et al. 2005b) or ‘above-ground hunting’ (Schöning et al. 2011). The genus *Anomma* was later demoted to the level of subgenus under *Dorylus* (Emery 1895).

However, not all species in the former subgenus *Anomma* are driver ants because Emery (1895) assigned additional, phylogenetically distinct, *Dorylus* species to the same subgenus (Kronauer et al. 2007). These latter species hunt in swarm raids that are restricted to the leaf-litter and do not ascend into the vegetation (“intermediate species” or “leaf-litter foraging species”; see Schöning et al. 2005b; Kronauer et al. 2007). While the true driver ant species form a clade, the intermediate species are more closely related to the subgenus, *Dorylus* s. str. (Kronauer et al. 2007). Since the subgeneric classification of *Dorylus* (Emery 1895) did not properly reflect phylogenetic relationships, it was subsequently abandoned in favour of informal species groups, so the true driver ants are now collectively referred to as the *Dorylus nigricans* group (Borowiec 2016).

At the same time, the species-level taxonomy and nomenclature of driver ants is in serious disarray. The taxonomy of the former subgenus *Anomma* was revised by Emery (1895, 1910), Santschi (1912), and Raignier and Van Boven (1955), but the status of the numerous infraspecific taxa and the identity of taxa described from a single sex or worker caste (see Bolton 2026) remain unclear. Consequently, we currently lack reliable identification keys. A plethora of problems in driver ant taxonomy is linked to the name *D. nigricans* Illiger, 1802, which was described on the basis of male specimens collected in Sierra Leone. Subsequently, no less than eighteen subspecific and infrasubspecific taxa were described or at some point subsumed under this name (Table 1). These difficulties essentially arise from four sources: (1) worker polymorphism, (2) intra- vs interspecific variation, (3) lack of unambiguous associations between males, queens, and workers, and (4) possible hybridisation.

**Table 1.** List of *Dorylus* taxa described or at some point subsumed under *D. nigricans*. Names in bold are formally treated in this paper; the other taxa need to be considered in a more comprehensive revision of the species group.

**Original description**	**Country of type locality**	**Based on which caste (male, queen, worker)**?	**Status (prior to this study) according to AntCat (Bolton 2026)**
***Anomma arcens* Westwood, 1847**	Liberia	worker	subspecies of *D. nigricans*
***Anomma burmeisteri* Shuckard, 1840**	Sierra Leone	worker	subspecies of *D. nigricans*
*Anomma molesta* Gerstäcker, 1859	Mozambique	worker	subspecies of *D. nigricans*
***Anomma pubescens* Roger, 1861**	Liberia	worker	junior synonym of subspecies *arcens* of *D. nigricans*
*Anomma rubella* Savage, 1849	Gabon	worker	subspecies of *D. nigricans*
Anomma sjostedti var. sjostedtiwilverthi Wasmann, 1917	Cameroon	worker	subspecies of *D. nigricans*
*Dorylus antinorii* Emery, 1881	Ethiopia	worker	junior synonym of subspecies *D. nigricans molestus*
Dorylus (Anomma) nigricans var. funereus Emery, 1895	Ghana	male	species (*D. funereus*)
Dorylus (Anomma) funereus acherontus Santschi, 1937	Republic of the Congo	male	subspecies of *D. funereus*
Dorylus (Anomma) funereus pardus Santschi, 1937	Democratic Republic of the Congo	male	subspecies of *D. funereus*
Dorylus (Anomma) funereus stygis Santschi, 1937	Democratic Republic of the Congo	male	subspecies of *D. funereus*
Dorylus (Anomma) funereus zumpti Santschi, 1937	Cameroon	male	subspecies of *D. funereus*
***Dorylus nigricans* Illiger, 1802**	Sierra Leone	male	species
***Dorylus nigricans burmeisteri hybrida* Santschi, 1912**	Senegal	worker	unavailable name
*Dorylus nigricans burmeisteri ornata* Santschi, 1912	Cameroon	worker	unavailable name
Dorylus (Anomma) burmeisteri var. pallida Santschi, 1921	Cameroon	worker	subspecies of *D. nigricans*
Dorylus (Anomma) nigricans subsp. sjostedti var. rufescens Wasmann, 1904	Cameroon	worker	species (*D. rufescens*)
Dorylus nigricans subsp. sjostedti Emery, 1899	Cameroon	worker	subspecies of *D. nigricans*
Dorylus (Anomma) nigricans var. terrificus Santschi, 1923	Democratic Republic of the Congo	worker	subspecies of *D. nigricans*

### (1) Worker polymorphism

**Figure 3.**
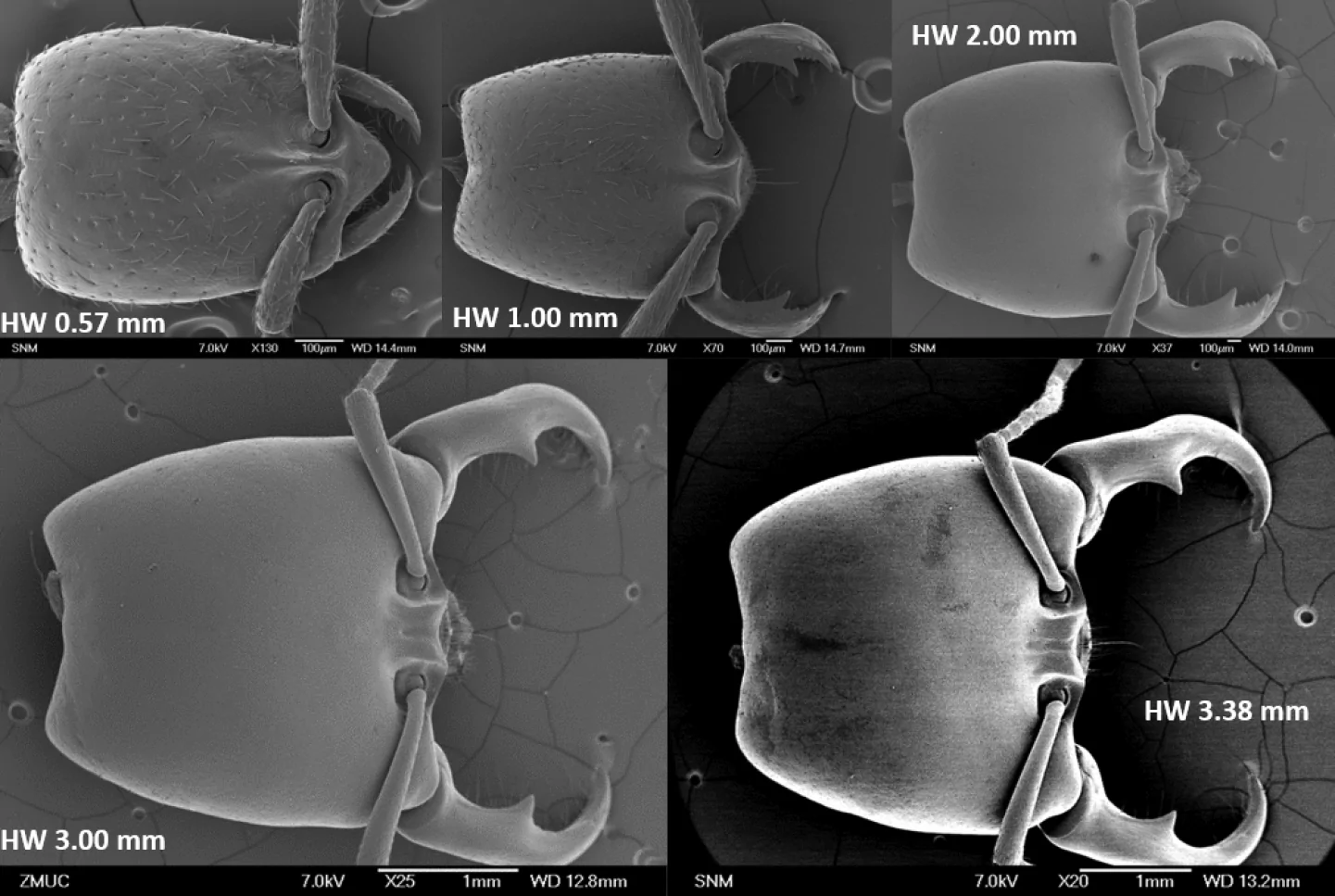
Scanning electron microscope pictures of driver ant workers collected from a single colony in Chogoria, Mount Kenya, Kenya. Note the differences in head shape, hairiness/pubescence, and mandible structure. The colony belongs to the East African Dorylus nigricans subspecies molestus (Gerstäcker, 1859). It appears to be a distinct species, but its status will need to be clarified elsewhere. Abbreviation: HW = head width.

**Figure 4.**
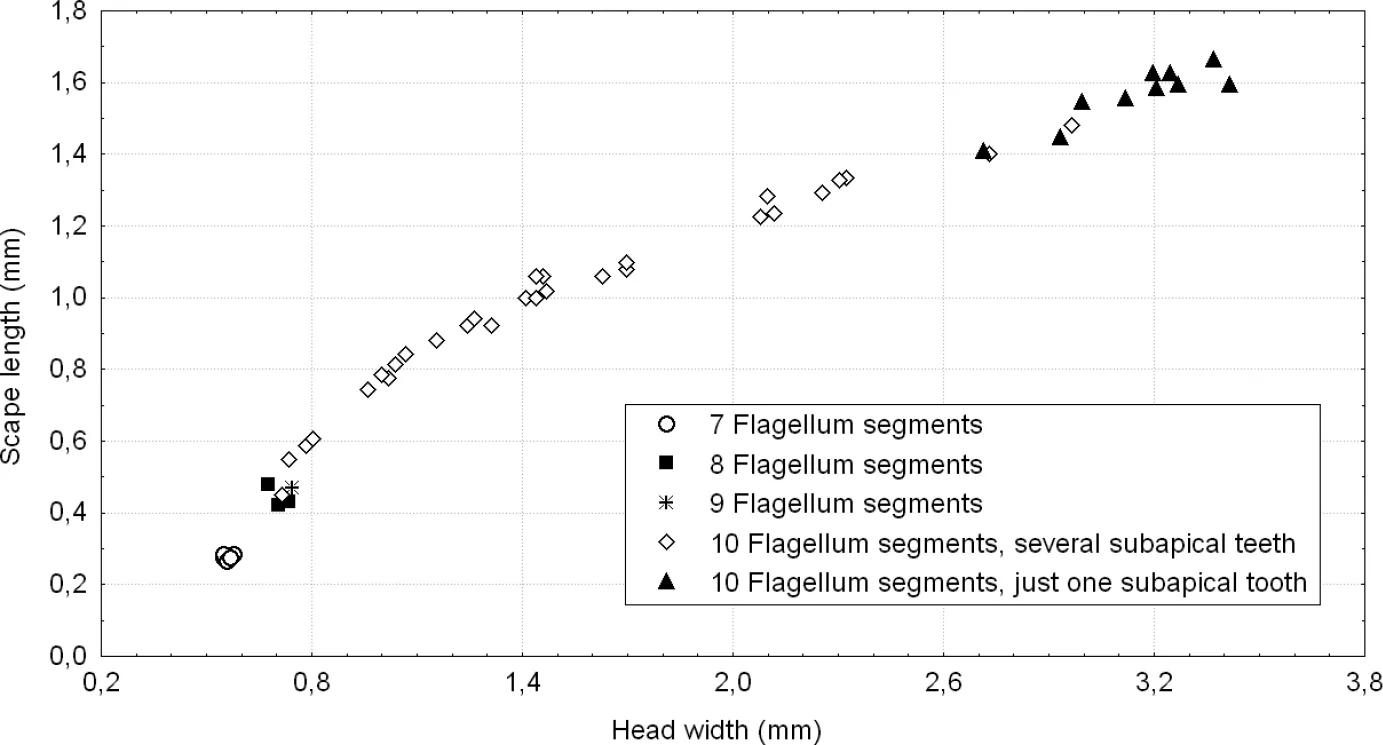
Antennal scape length in relation to head width in 51 driver ant workers collected from the same colony as in Fig. 3. The relationship resembles a diphasic or curvilinear allometry (Wills et al. 2018).

With the genera *Atta*, *Eciton*, and *Carebara*, *Dorylus* displays the most extreme form of worker polymorphism among the ants (Hölldobler and Wilson 1990). Within a single driver ant colony, worker head width may vary more than six-fold (range 0.55–3.7 mm in *D. wilverthi*) and individual dry mass by a factor in excess of 160 (Schöning et al. 2005b). Over this extensive size range, workers also differ in dramatic ways in antennal segmentation (number of flagellomeres varies from seven to ten), mandibular dentition, head shape, clypeus structure, hairiness/pubescence, and other features (Figs 3, 4; see also Emery 1901; Raignier and Van Boven 1955; Cohic 1948; Hollingsworth 1960; Raignier et al. 1974). It is therefore fair to say that a single driver ant colony harbours worker morphs that display a greater morphological diversity than some entire ant genera.

This extreme variation in morphology is correlated with extensive division of labour (Gotwald 1995; Franks et al. 2001; Schöning et al. 2005b). The smallest individuals (head width ~ 0.6 mm) usually take care of the queen and remain inside the nest except during nest relocations (Raignier et al. 1974), while the rare workers of the largest size class (> 2.5 mm head width) specialise in defensive tasks and play an important role in capturing, dismembering, and retrieving prey (Schöning et al. 2005b). The pronounced polymorphism complicates taxonomic studies as it renders the search for diagnostic characters difficult. It is thus not directly possible to test whether sets of worker specimens with non-overlapping size ranges are conspecific. For example, Emery (1881, 1901) erroneously described a small driver ant worker with eight flagellomeres as a new species in the subgenus *Alaopone*, where even the largest workers have only eight flagellomeres. Similarly, the strong pubescence of small workers misled Roger (1861) to describe these specimens as the new species *Anomma pubescens*, although the larger individuals had already been described by Westwood (1847) as *Anomma arcens* (Emery 1895). The examination of large series of workers encompassing a wide size range is the best way to avoid these unusual pitfalls resulting from the perplexing morphological diversity within driver ant colonies.

### (2) Intra- vs interspecific variation

The second challenge in driver ant taxonomy is to distinguish the formidable intraspecific variation from interspecific variation. Most of the infraspecific taxa (Bolton 2026) were described by Santschi, who often placed much value on the classification of single or a few individuals characterised by minute variation in coloration or other features without providing quantitative evidence to show that these specimens indeed represented a separate species (see also Brown 1955; Wehner 1983; Raczkowski and Wenzel 2007). The most effective remedy is to collect and quantitatively examine large worker samples from a wide geographic range to test whether a putatively diagnostic trait may in fact be variable within colonies, populations, or across populations in a region, and thus of little use.

### (3) Lack of associations between males, queens, and workers

Most taxa in the driver ant clade have been described based only on a single caste or sex. Four species (*atratus*, *funereus*, *nigricans*, *stanleyi*) are known only from the large and distinctive males, which disperse on the wing at night and are often collected only at lights. The crucially important association of males with conspecific workers (and queens) can be demonstrated in two ways (Schöning et al. 2008b). Ideally, males are found inside their natal nest (e.g., Forel 1909; Raignier and Van Boven 1955) or while flying off from it (e.g., Kronauer et al. 2004, 2006). In these cases, workers can be directly collected together with the males. In rare cases, very small workers from the natal nest can be found in the genital capsule of dispersing males (Emery 1895: 737; CS, pers. obs.). However, in such cases the link between the male and worker phenotypes is of little help because the smallest workers lack simple diagnostic features and can often not be identified at the species level (Emery 1895: 703). Alternatively, comparative analyses of DNA sequence data can elucidate the relationships between males found at light sources and putatively conspecific workers (Berghoff 2003; Ward 2007; Schöning et al. 2008b), given that intraspecific genetic variation is usually low compared to interspecific variation (e.g., Hebert et al. 2003).

### (4) Possible hybridisation

Finally, hybridisation between driver ants is known to occur in western Kenya (Kronauer et al. 2011; Butler et al. 2018) and may also be widespread elsewhere. Raignier (1972) reported finding heterospecific males in driver ant colonies and Wasmann (1916, 1917) described the “hybrid variety” Anomma sjostedti var. sjostedtiwilverthi. The occurrence of hybrid workers complicates the recognition of species-specific morphological characters and may also compromise the establishment of male-worker associations via DNA analysis when hybridisation makes species appear paraphyletic, as has been shown for mitochondrial data in East African driver ants (Kronauer et al. 2011).

In the present study, we sampled the diversity of West African driver ant species by extensive surveys of workers and examined the variation in qualitative and quantitative worker traits to determine species boundaries. We find that only three morphologically distinct driver ant species occur in this region. Since numerous forms of driver ants were described under the name *D. nigricans*, the correct male-worker association for this species is crucially important for clarifying the nomenclature of the driver ant species group. This insight emerged from the study by Kronauer et al. (2007), which showed that a male morphologically similar to one of the *D. nigricans* type specimens was genetically similar to workers identified as *D. arcens* and *D. burmeisteri*. We therefore scrutinised the possible male-worker association between these forms by analysing mitochondrial and nuclear DNA sequence data from males and workers of all three co-occurring driver ant species from the Taï National Park in Ivory Coast. We focussed on a single collection locality where the species occur in sympatry because such a modest geographic sampling scale often delivers the highest accuracy in DNA barcoding (Bergsten et al. 2012). Finally, we developed an identification key to the large workers of the three species in the region and described hitherto unknown queen castes.

## Materials and methods

### Surveys of driver ant species

This study was made possible by collaborative efforts with primatologists studying insectivory by chimpanzees (e.g., Möbius et al. 2008; Schöning et al. 2008c; Sanz et al. 2010; Vaidyanathan 2011; Luncz and Boesch 2015). In the course of the extensive field studies undertaken to elucidate whether geographic variation in chimpanzee insectivory is linked to environmental factors and/or reflects cultural differences among chimpanzee communities, we obtained data and large numbers of worker samples from 21 sites (Suppl. material 6) from Senegal to Ghana. Driver ant worker samples were collected from nests or columns encountered during opportunistic or transect walks. Driver ant workers can be distinguished from other *Dorylus* workers by the presence of basisternal spines on the ventral side of the mesosoma (Gotwald and Schaefer 1982). An examination of this trait in all species used in the study by Kronauer et al. 2007 showed its diagnostic value for recognising workers of the driver ant clade (data not shown). At some sites chimpanzee faecal samples were additionally examined for the presence of driver ant fragments. At Taï National Park and Bobiri, *Dorylus* males were collected during nights at artificial lights. Specimens were stored in 70% or 100% ethanol. Driver ant males were later sorted from these samples using the identification key developed by Gotwald (1982) and additionally the diagnostic traits dark colouration, relatively long head (HL / HW > 0.59), and the ratio between prong length and distance between prong apices (PL / PD > 1.8), since these traits had turned out to be synapomorphies of males of the driver ant clade in an examination of all the species included in the study by Kronauer et al. (2007) (data not shown).

### Morphological analyses of workers, males, and queens

We examined more than 1000 driver ant worker samples, 18 driver ant males, two driver ant queens, and associated workers from the same nests as well as the type specimens for all relevant names of West African driver ants except for *Anomma burmeisteri* Shuckard, 1840, the type specimen of which is apparently lost (James Hogan, George Else and Suzanne Ryder, pers. comm. 18 March 2005 and 11 February 2026), *Dorylus atratus* Smith, 1859, the type specimen of which is apparently likewise lost (George Else, pers. comm. 23 January 2006), and *Dorylus funereus* Emery, 1895. For the latter species, images of the type specimen provided from AntWeb (2025) (https://www.antweb.org/specimen.do?code=casent0903695) were used for a comparison with *Dorylus nigricans* males.

Entomological collections cited in acronym form refer to the following sources:

**CSAC** Caspar Schöning personal collection, Görlitz, Germany;

**MCZC** Museum of Comparative Zoology, Harvard University, Cambridge, USA;

**NHMB** Naturhistorisches Museum Basel, Basel, Switzerland;

**NHMD** Natural History Museum Denmark, University of Copenhagen, Denmark;

**OUMNH** Hope Entomological Collections, University Museum, Oxford, UK;

**RMCA** Royal Museum for Central Africa, Tervuren, Belgium;

**SMNG** Senckenberg Museum für Naturkunde, Görlitz, Germany;

**ZMHB** Museum für Naturkunde der Humboldt-Universität, Berlin, Germany.

**Figure 5.**
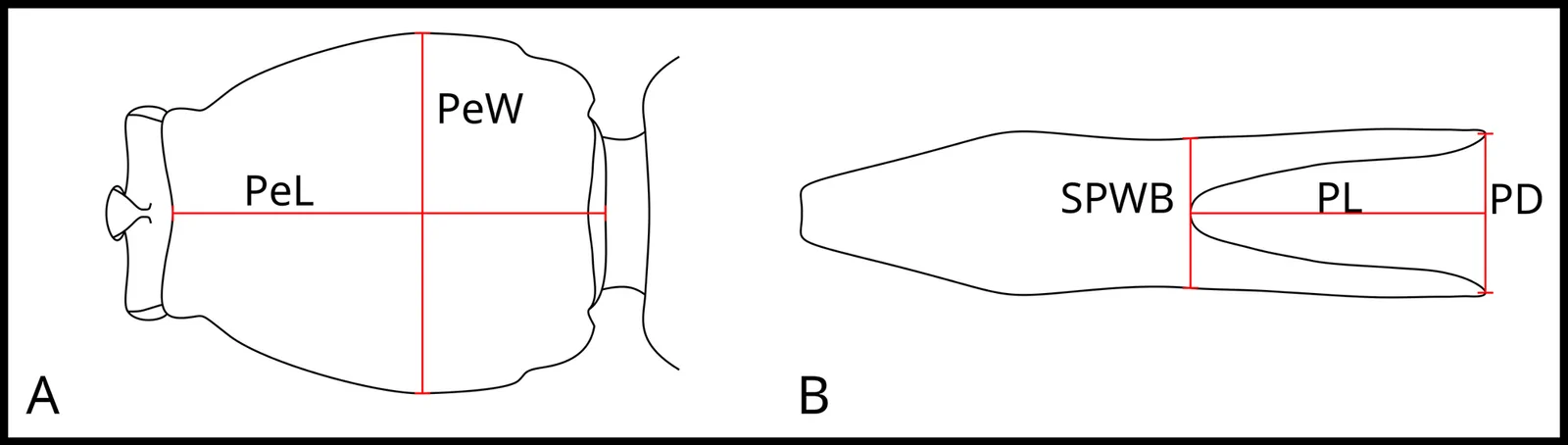
Visualisation of some measurements. **A**. Dorsal view of the petiole; **B**. Dorsal view of the subgenital plate. Abbreviations: PeW = maximum petiole width in dorsal view; PeL = maximum length of the petiole in dorsal view; PD = distance between the apices of the subgenital plate prongs. PL = prong length of the subgenital plate, measured in a straight line parallel to the longitudinal axis of the subgenital plate from the apical point of the right prong to its base; SPWB = width of the subgenital plate at the baseline of the prongs.

All observations were made at magnifications 6–288× on a Leica MS 5 or a Leica M165 C microscope. We examined a suite of morphological characters commonly used in ant systematics such as pilosity, pubescence, colour, shape of the head, shape of the petiole and mandibular dentition. In addition, we quantified several standard morphometric characters using an ocular micrometre. Since the smaller *Dorylus* workers are less differentiated between species (Schöning et al. 2005b) and subcastes can only be categorised in an arbitrary manner (see Fig. 2), we decided to use only workers with a HW larger than an arbitrary cut-off value of 1.5 mm for the morphometric analyses. We took measurements for up to six individuals per sample. The characters examined were:

**PW** maximum width of pronotum in dorsal view;

**HW** maximum measurable head width in frontal view (including the eyes in males);

**HL** head length, measured in a straight line from the anteriormost point of the median clypeal margin to the mid-point of the posterior head margin (in males including the ocelli);

**SL** maximum straight line scape length excluding the basal condyle;

**HFL** hind femur length;

**HTL** hind tibia length;

**PeW** maximum petiole width in dorsal view (Fig. 5);

**PeL** maximum length of the petiole in dorsal view (Fig. 5).

For the queens we also measured:

**PropW** maximum propodeum width in dorsal view;

**TL** total length.

Additionally, the following characters were examined in males:

**CL** diagonal length of the submarginal cell of the forewing;

**DPF** distance between the anteriormost points of the parapsidal furrows;

**EL** maximum length of the eye in lateral view;

**MHairs** conspicuous hairs on the mesosoma being present (1) or absent (0);

**MW** maximum mesosoma width in dorsal view;

**PD** distance between the apices of the subgenital plate prongs;

**SPWB** width of the subgenital plate at the baseline of the prongs;

**PL** prong length of the subgenital plate, measured in a straight line parallel to the longitudinal axis of the subgenital plate from the apical point of the right prong to its base.

Several specimens were imaged with an Automontage system (www.syncroscopy.com) by April Nobile at the California Academy of Sciences and images are available online as part of the AntWeb database (http://www.antweb.org). Additional Automontage images were taken at the Senckenberg Museum for Natural History in Görlitz with a Keyence VHX 7000, while SEM pictures were taken at the Zoological Museum of the University of Copenhagen (now part of the Natural History Museum Denmark). All statistical analysis were conducted, and graphs were produced in R 4.5.1 (R Core Team 2025). The corresponding script is provided in the Suppl. material 7.

### Molecular assessment of species boundaries and male-worker matching

We included five male specimens morphologically similar to one of the *D. nigricans* type specimens, four worker specimens morphologically similar to the *Anomma arcens* type specimens, five worker specimens later identified as *D. burmeisteri*, and seven worker specimens of *D. mayri* (Table 2). All individuals were collected from separate colonies at Taï National Park.

**Table 2.** List of male and worker specimens from Taï National Park from which sequence data were obtained. For each individual we list the GenBank accession numbers of the three gene sequences. “Pseudogene” indicates the amplification of a putative nuclear mitochondrial (numt) pseudogene. All specimens are kept in SMNG.

**Form**	**Label**	**Collector**	**GPS coordinates in WGS84**	**Species**	***LW*-*Rh***	***wnt*-*1***	***COII***	**Unique specimen identifier**
Male	Taï NP Südcamp May / June 2006 Male C, MM26	Yasmin Möbius	5.83896, -7.32056	*D. nigricans*	PZ 495383	PZ 495362	PZ 495324	SMNG-COT-2006-026-01
Male	Taï NP Südcamp May / June 2006 Male D, MM27	Yasmin Möbius	5.83896, -7.32056	*D. nigricans*	PZ 495384	PZ 495363	PZ 495325	SMNG-COT-2006-027-01
Male	Taï NP Südcamp May / June 2006 Male E, MM28	Yasmin Möbius	5.83896, -7.32056	*D. nigricans*	PZ 495385	PZ 495364	PZ 495326	SMNG-COT-2006-028-01
Male	Taï NP Südcamp May / June 2006 Male F, MM29	Yasmin Möbius	5.83896, -7.32056	*D. nigricans*	PZ 495386	PZ 495365	PZ 495327	SMNG-COT-2006-029-01
Male	Taï NP Südcamp May / June 2006 Male G, MM30	Yasmin Möbius	5.83896, -7.32056	*D. nigricans*	PZ 495387	PZ 495366	PZ 495328	SMNG-COT-2006-030-01
Worker	T40, 18 May 2006, MM31	Caspar Schöning	5.83664, -7.32841	*D. nigricans*	PZ 495388	PZ 495367	PZ 495329	SMNG-COT-2006-031-01
Worker	Lydia 32, 04 July 2010, MM38	Lydia Luncz	5.86996, -7.33837	*D. nigricans*	PZ 495389	PZ 495368	PZ 495330	SMNG-COT-2010-038-01
Worker	Lydia 35, 05 July 2010, MM39	Lydia Luncz	5.85944, -7.32983	*D. nigricans*	PZ 495390	PZ 495369	PZ 495331	SMNG-COT-2010-039-01
Worker	Lydia 38, 11 July 2010, MM40	Lydia Luncz	5.82245, -7.31768	*D. nigricans*	PZ 495391	PZ 495370	PZ 495332	SMNG-COT-2010-040-01
Worker	Lydia 21, 25 May 2010, MM46	Lydia Luncz	5.83505, -7.29284	*D. burmeisteri*	PZ 495392	PZ 495371	PZ 495333	SMNG-COT-2010-046-01
Worker	Lydia 27, 02 June 2010, MM47	Lydia Luncz	5.86364, -7.35215	*D. burmeisteri*	PZ 495393	PZ 495372	PZ 495334	SMNG-COT-2010-047-01
Worker	Lydia 27, 12 March 2010, MM48	Lydia Luncz	5.85, -7.15	*D. burmeisteri*	PZ 495394	PZ 495373	PZ 495335	SMNG-COT-2010-048-01
Worker	Lydia 51, 20 March 2010, MM49	Lydia Luncz	5.85, -7.15	*D. burmeisteri*	PZ 495395	PZ 495374	PZ 495336	SMNG-COT-2010-049-01
Worker	Lydia 42, 24 Oct 2010, MM50	Lydia Luncz	5.85, -7.15	*D. burmeisteri*	PZ 495396	PZ 495375	PZ 495337	SMNG-COT-2010-050-01
Worker	T26, 13 May 2006, MM55	Caspar Schöning	5.80512, -7.30495	*D. mayri*	PZ 495397	PZ 495376	Pseudogene	SMNG-COT-2006-055-01
Worker	T20, 12 May 2006, MM56	Caspar Schöning	5.81799, -7.31686	*D. mayri*	PZ 495398	PZ 495377	Pseudogene	SMNG-COT-2006-056-01
Worker	T6, 07 May 2006, MM57	Caspar Schöning	5.80457, -7.31409	*D. mayri*	PZ 495399	PZ 495378	Pseudogene	SMNG-COT-2006-057-01
Worker	T37, 16 May 2006, MM58	Caspar Schöning	5.82084, -7.30575	*D. mayri*	PZ 495400	PZ 495379	Pseudogene	SMNG-COT-2006-058-01
Worker	Lydia 29, 27 June 2010, MM59	Lydia Luncz	5.85, -7.15	*D. mayri*	PZ 495401	PZ 495380	Pseudogene	SMNG-COT-2010-059-01
Worker	Lydia 36, 06 July 2010, MM60	Lydia Luncz	5.86508, -7.32565	*D. mayri*	PZ 495402	PZ 495381	Pseudogene	SMNG-COT-2010-060-01
Worker	Lydia 37, 2009, LL37	Lydia Luncz	5.85, -7.15	*D. mayri*	PZ 495403	PZ 495382	Pseudogene	SMNG-COT-2009-137-01

We sequenced fragments of the mitochondrial gene *cytochrome oxidase subunit II* (*COII*, total length 619 bp, coding sequence (CDS) 575 bp), as well as the nuclear genes *wingless* (*wnt*-*1*, 400 bp) and *long*-*wavelength rhodopsin* (*LW*-*Rh*, total length 535 bp with two CDSs with 226 and 223 bp, respectively). DNA was extracted from ant legs using the QIAGEN (Valencia, CA, USA) DNeasy kit. The *COII* fragment was amplified by standard polymerase chain reaction (PCR) using the primers tRNALeu (Kronauer et al. 2007) and Barbara (Simon et al. 1994) with an annealing temperature of 45 °C. The *wnt*-*1* and *LW*-*Rh* fragments were amplified with primers Wg503F (Schultz and Brady 2008) and Wg1032R (Abouheif and Wray 2002), and LR143F and LR639ER (Ward and Downie 2005), respectively, in both cases with an annealing temperature of 52 °C. The PCR cocktail contained 15.05 µL ddH_2_O, 2.5 µL 10× PCR Buffer, 1 µL 25 mM MgCl_2_, 0.25 µL dNTPs (25 mM each), 2 µL of each primer (10 ×), 0.2 µL QIAGEN Taq DNA Polymerase, and 2 µL DNA extract. PCR products were purified and Sanger sequenced in both directions by the company Macrogen.

Unique sequences were deposited in NCBI GenBank (accession numbers PZ495324 – PZ495337 and PZ495362 – PZ495403, see Table 2). As an outgroup, we used the closely related non-driver ant *Dorylus helvolus* [GenBank accession numbers: EF413794 (*COII*), AY867427 (*wnt*-*1*), AY867489 (*LW*-*Rh*)]. We examined species boundaries based on the obtained molecular data using two approaches following von Beeren et al. (2016). We constructed maximum likelihood (ML) phylogenies and estimated pairwise distances separately for nuclear (nDNA) and mitochondrial (mtDNA) DNA using the MEGA 12 software (Kumar et al. 2024). We included all three codon positions of a CDS and ignored non-coding sites (Codon Starts: *COII* – 1^st^ site, *LW*-*Rh* – CDS_1: 2^nd^ site, CDS_2: 1^st^ site, *wnt*-*1*: 2^nd^ site). The best nucleotide substitution model was selected based on Bayesian Information Criterion (BIC) scores in MEGA12 using the Kimura (1980) two-parameter model for nuclear sequences and the Tamura (1992) model for *COII*. Support for each node was assessed via 1000 bootstrapping iterations. Cases where sympatric specimens clustered in separate clades based on mtDNA and display fixed diagnostic variants at nuclear loci, with no shared alleles between taxa, support the recognition of distinct species in the present dataset. In a second approach, we compared the intra- and interspecific distributions of pairwise distances for mtDNA and nDNA separately using R 4.5.1 (R Core Team 2025). Inkscape (https://inkscape.org/) was used to edit trees and other figures for display purposes.

## Results

### Surveys of driver ant workers

**Figure 6.**
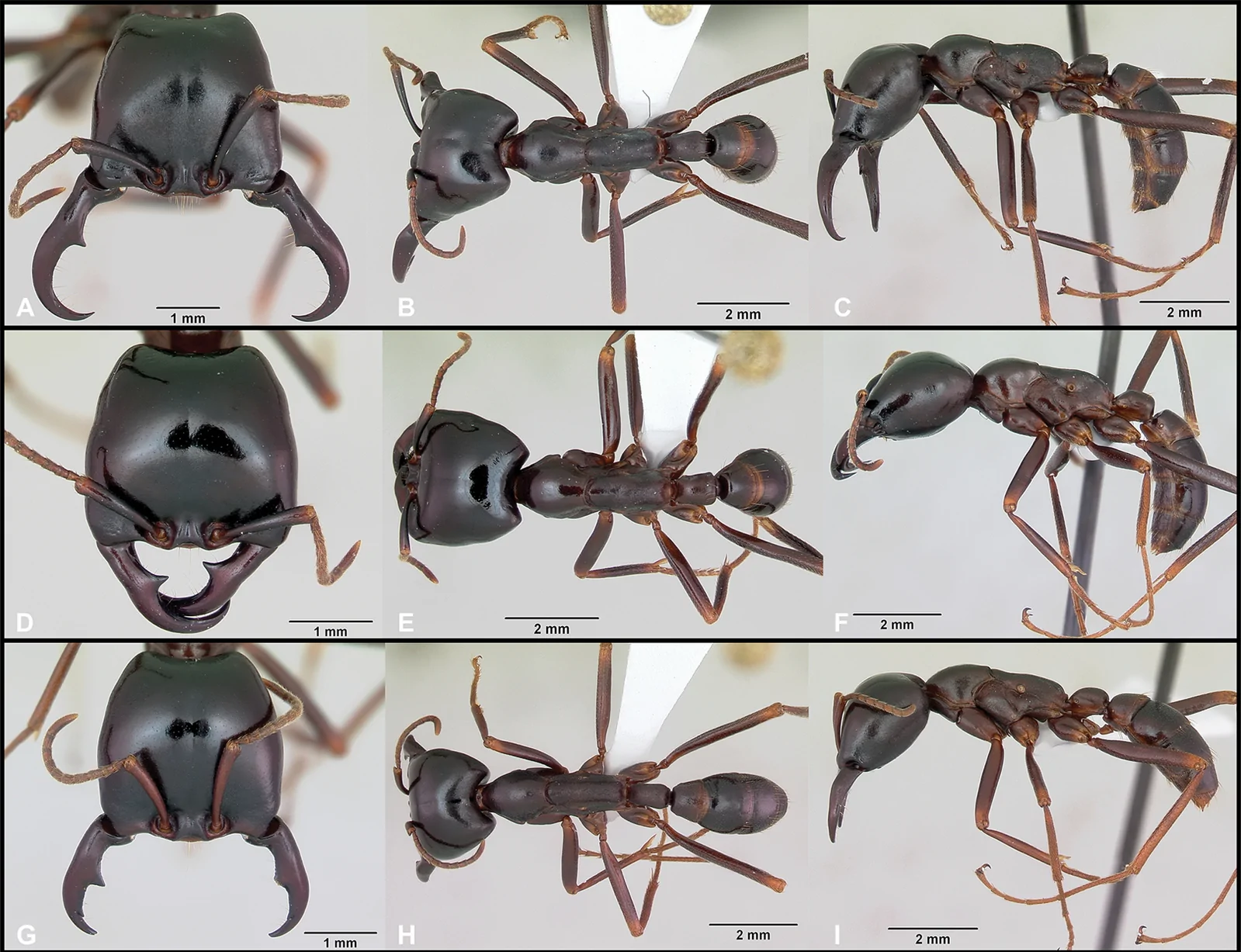
Workers of West African driver ant species in full-face (**A, D, G**), dorsal (**B, E, H**), and lateral (**C, F, I**) view. From top to bottom. **A–C**. *Dorylus nigricans* (CASENT0172642, Ivory Coast, Taï National Park, leg. C. Schöning, May 6, 2006); **D–F**. *D. burmeisteri* (CASENT0172651, Ivory Coast, Taï National Park, leg. C. Schöning, May 10, 2006); **G–I**. *D. mayri* (CASENT0172639, leg. C. Schöning, Taï National Park, May 13, 2006). All images are by April Nobile from AntWeb.

**Figure 7.**
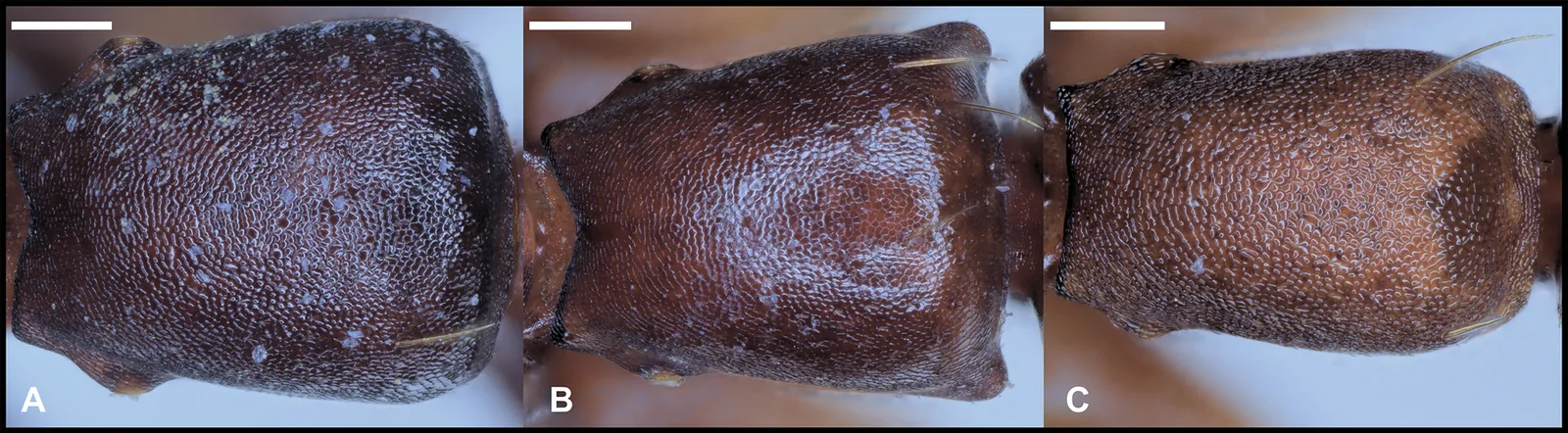
Petiole shape of representative driver ant workers in dorsal view. From left to right. **A**. *Dorylus nigricans* (specimen SMNG-GHA-2006-174-01); **B**. *D. burmeisteri* (specimen SMNG-GHA-2006-160-01); **C**. *D. mayri* (specimen SMNG-CON-2009-166-01). The scale bar represents 0.2 mm in all three images.

The surveys yielded only three species of driver ants in the region from Senegal to Ghana, and all of them conformed to workers of already described taxa. At Boe, Bossou, Grebo, Nimba Mountains, Sobeya, and Taï the sample sizes were so large (> 30) that we concluded that sampling was complete enough to infer that we had covered the total number of species occurring in the area. The three species can be distinguished by differences in head shape, head sculpture, petiole shape, and the relative length of antennae and legs (Figs 6, 7).

**Figure 8.**
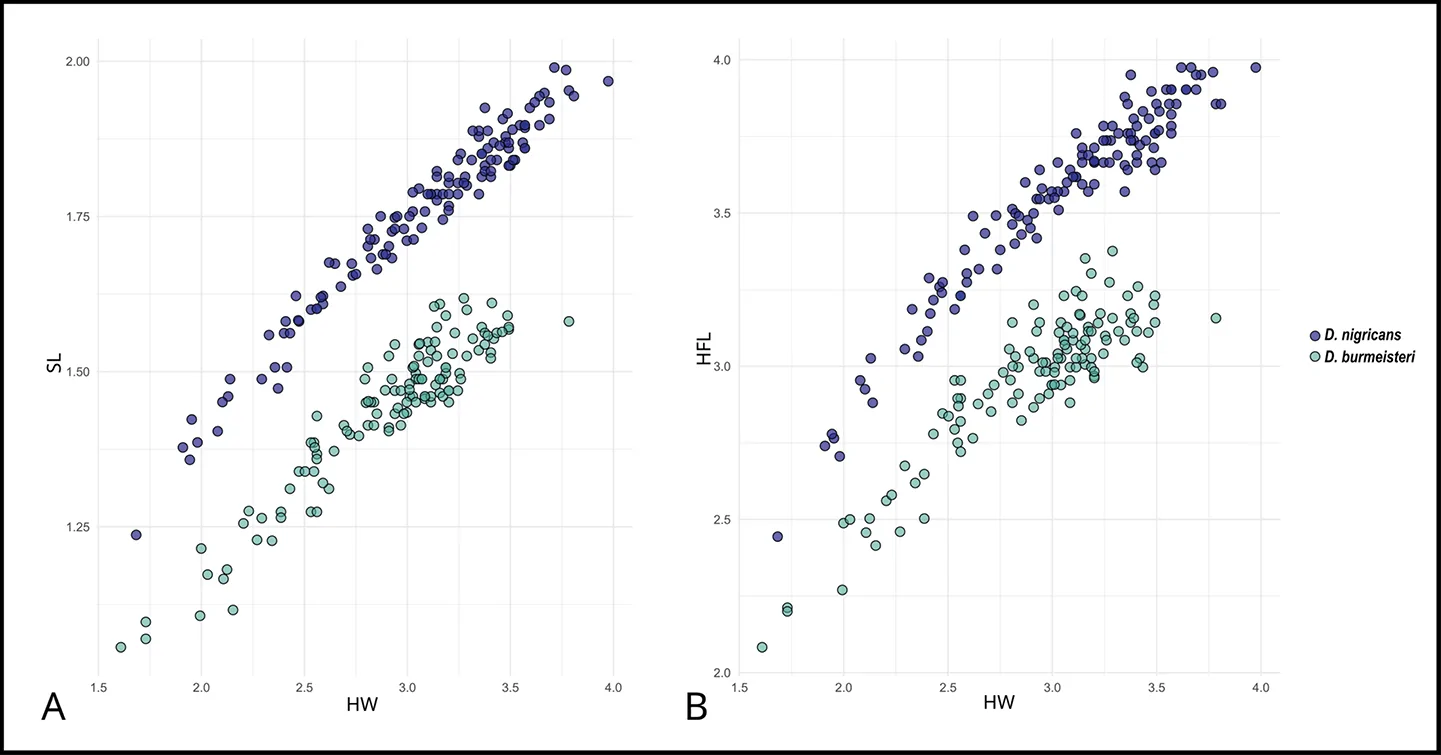
Morphometric measurements of large workers of *Dorylus burmeisteri* and *D. nigricans*; x-axis: head width in mm (HW), y-axis. **A**. Scape length in mm (SL); **B**. Hind femur length in mm (HFL); blue: (*n* = 127), green: (*n* = 123).

**Figure 9.**
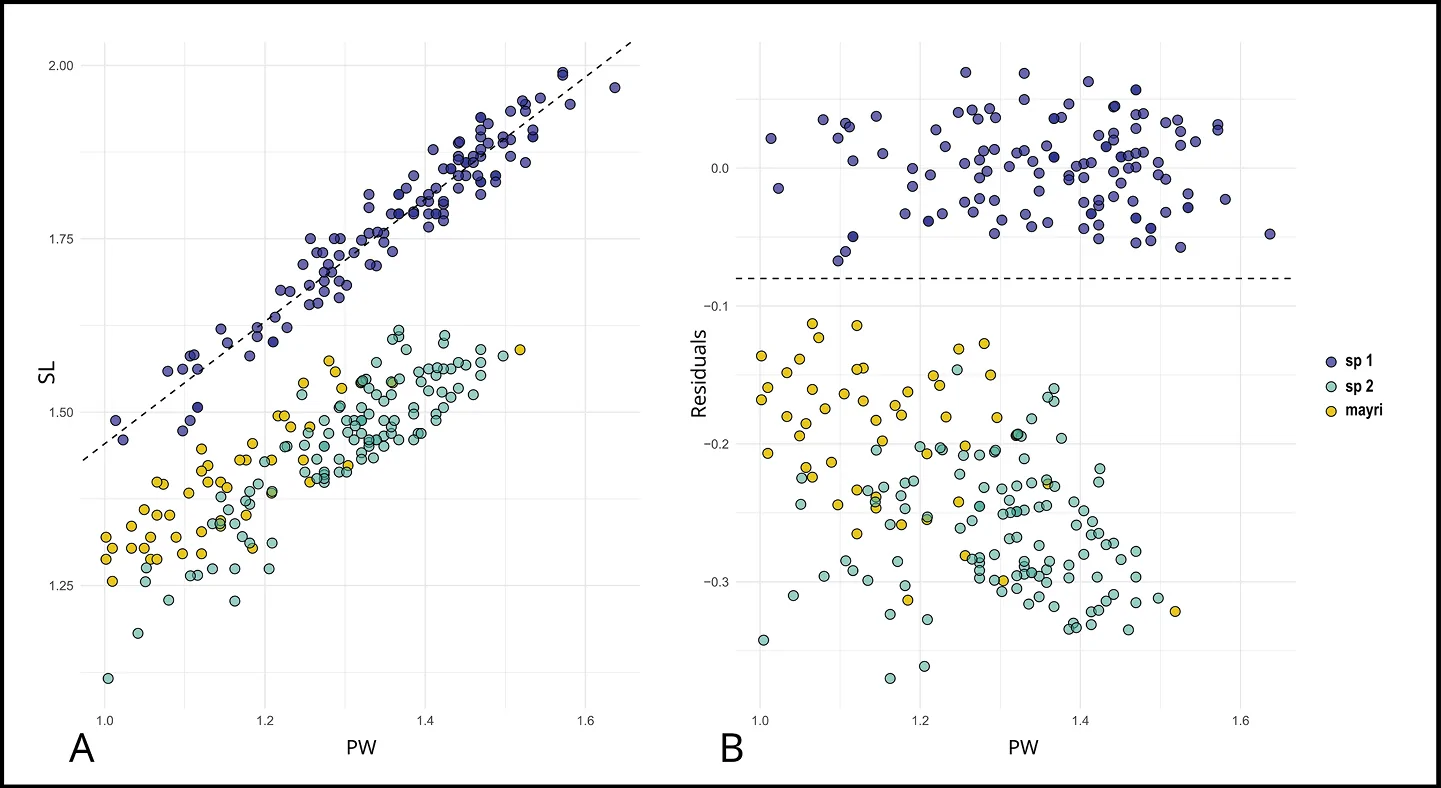
Morphometric measurements of large workers of *Dorylus burmeisteri*, *D. mayri* and *D. nigricans*. **A**. Scape length (SL in mm) in relation to pronotum width (PW in mm) of large workers (HW > 1.5 mm) of West African driver ants; **B**. Residuals of a linear regression on SL ~ a + b * PW; for *D. nigricans* (dotted line in A) a = 0.5728, b = 0.8816. Symbol colours: *Dorylus nigricans* – blue (*n* = 127), *D. burmeisteri* – green (*n* = 123); *D. mayri* – yellow (*n* = 63). The dashed horizontal line is placed at a residual of -0.08 mm.

The workers that correspond to specimens originally described under the name *Anomma arcens* Westwood, 1847 and were later identified as *D. nigricans* (see below) have longer antennae and legs than specimens that conform to the workers originally described under the name *A. burmeisteri* Shuckard, 1840 and that were later named *D. burmeisteri* (Figs 8, 9).

**Figure 10.**
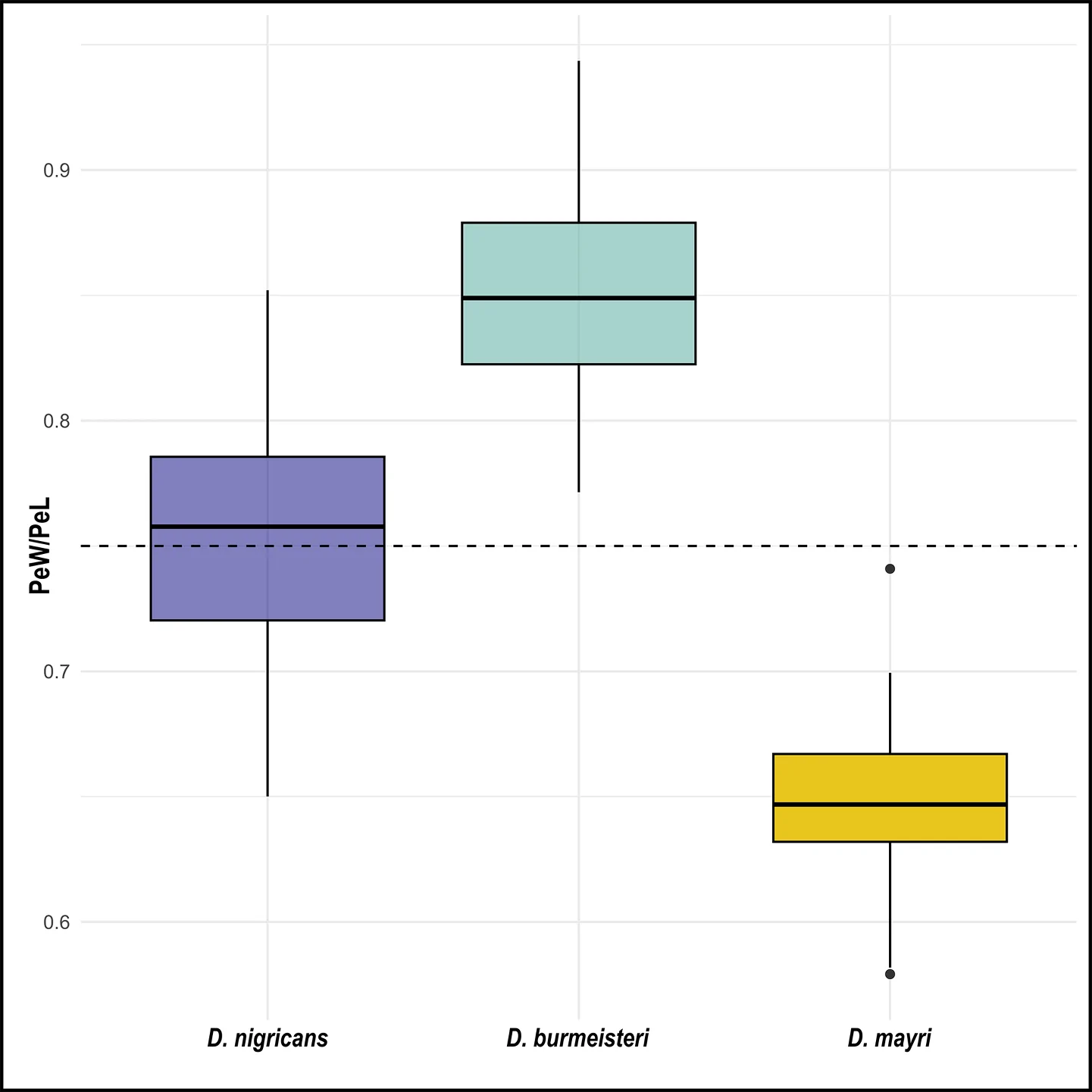
Shape of the petiole in large workers of West African driver ant species (HW > 2.0 mm); y-axis: petiole width (PeW) divided by petiole length (PeL), dashed horizontal line is placed at 0.75, blue: *Dorylus nigricans* (*n* = 50), green: *D. burmeisteri* (*n* = 52), yellow: *D. mayri* (*n* = 50).

*Dorylus nigricans* workers can also be distinguished by the pronounced reticulate microsculpture visible in full face view of the head, which causes a dull appearance (Fig. 6). The workers of the third species, *D. mayri*, are rather distinct from the two others because of the round rather than trapezoidal head shape of large workers (Fig. 6), the narrow, almost rectangular petiole in dorsal view (Figs 7, 10) and the rarity of large workers (HW > 3.00 mm) with only one subapical mandibular tooth. The maximum head width among the specimens measured in this study was only 3.2 mm in *D. mayri* compared to 3.97 mm in *D. nigricans* and 3.78 mm in *D. burmeisteri*.

### Examination of *D. nigricans* type specimens and other West African driver ant males

In the ZMHB collection there are two male specimens labelled as *D. nigricans* Illiger, henceforth referred to as syntypes A and B. Both are in poor condition. Some appendages are broken off, and body parts of specimen B are covered with glue (or similar material). We describe the two specimens in detail below.

#### Syntype A

**Figure 11.**
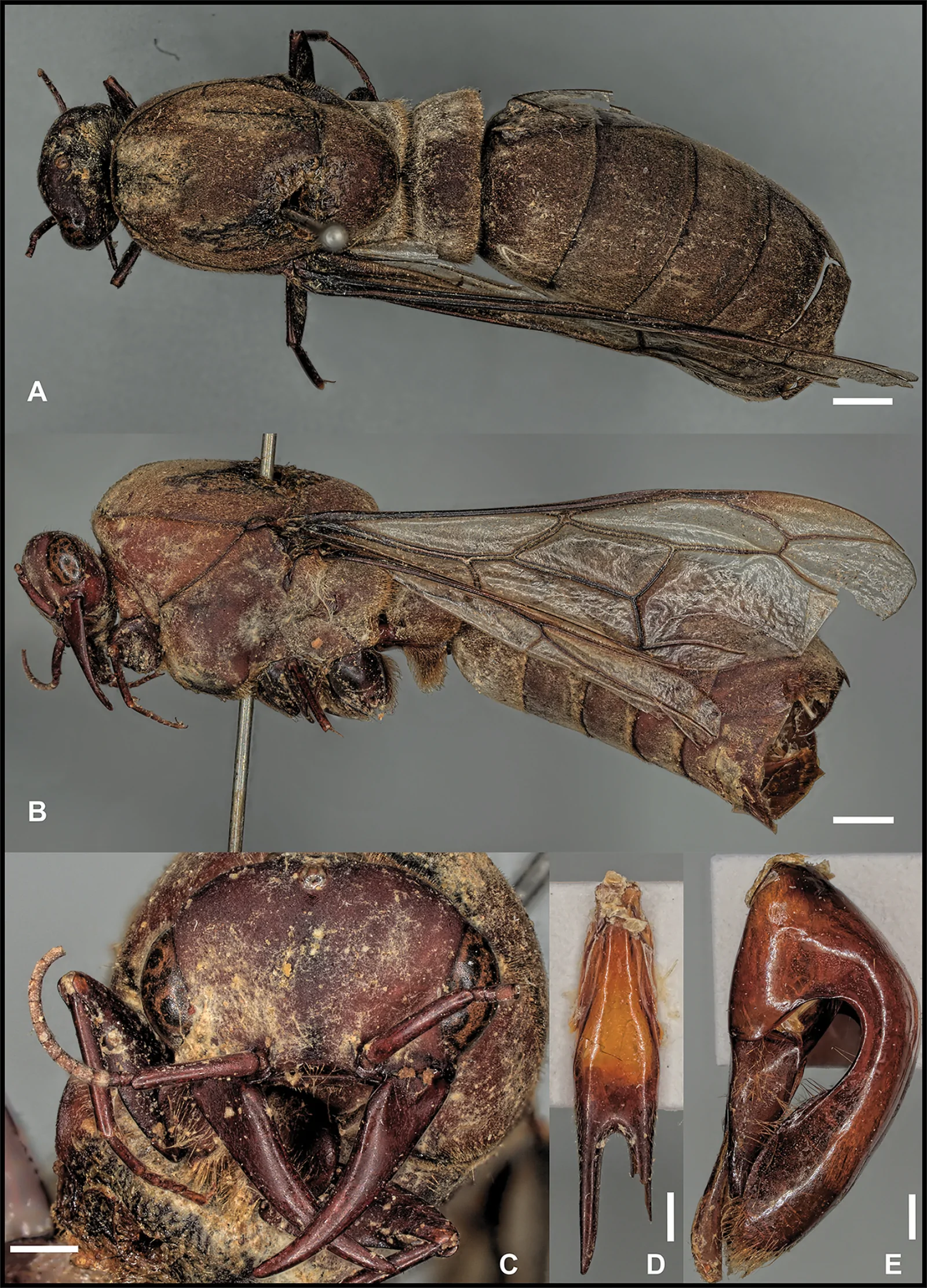
Syntype male specimen A of *Dorylus nigricans* in ZMHB. **A**. Dorsal overview; **B**. Lateral overview; **C**. Head in full-face view; **D**. Subgenital plate (**D**); **E**. Genital capsule. Scale bars: 2 mm (**A, B**); 1 mm (**C–E**). PL and PD were measured when the specimens were originally examined in 2006. When pictures were taken in 2025, the subgenital plate had been damaged. This specimen was designated as lectotype.

**Figure 12.**
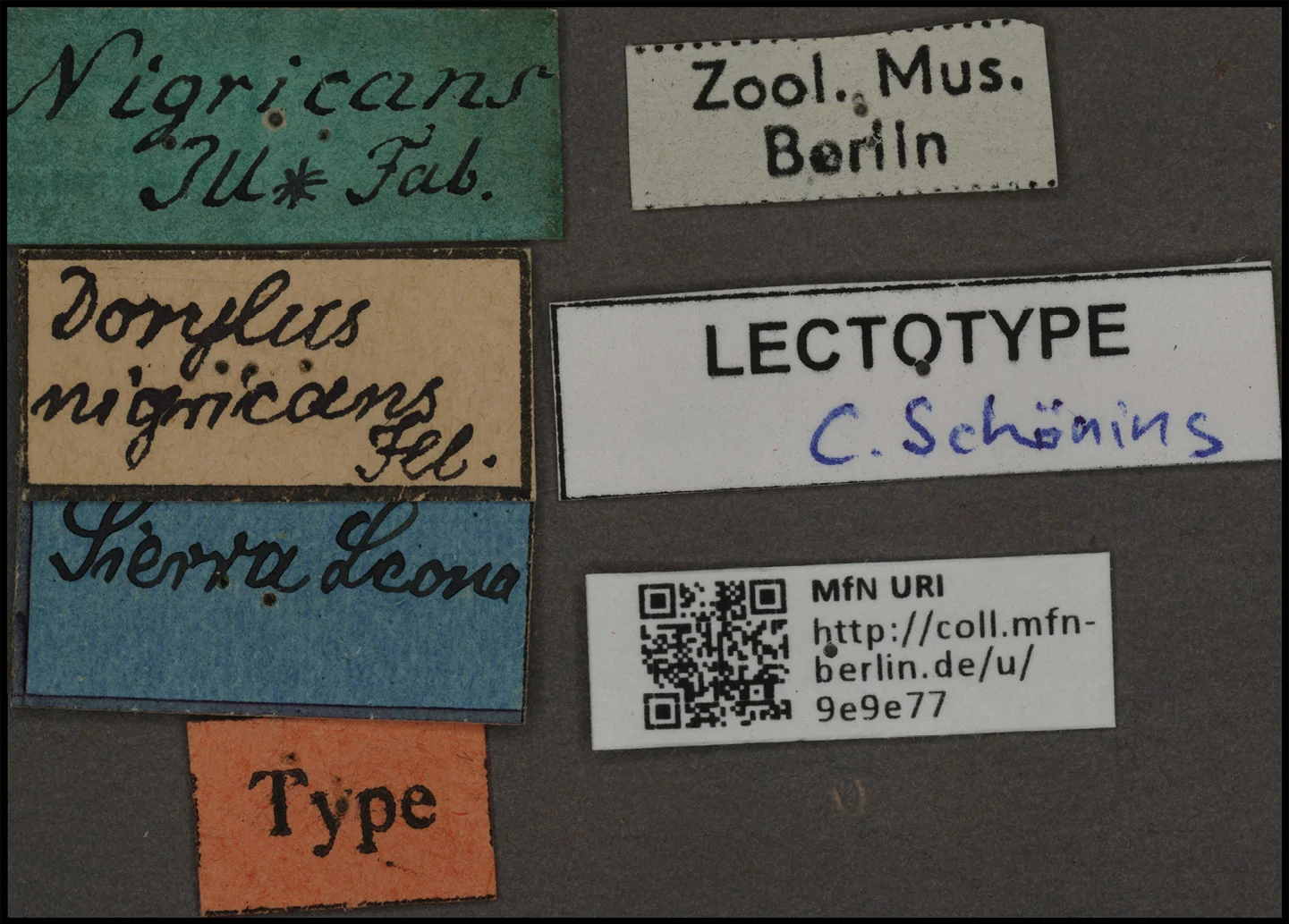
Labels of *Dorylus nigricans* syntype specimen A (Fig. 11) in ZMHB, designated as lectotype.

**Figure 13.**
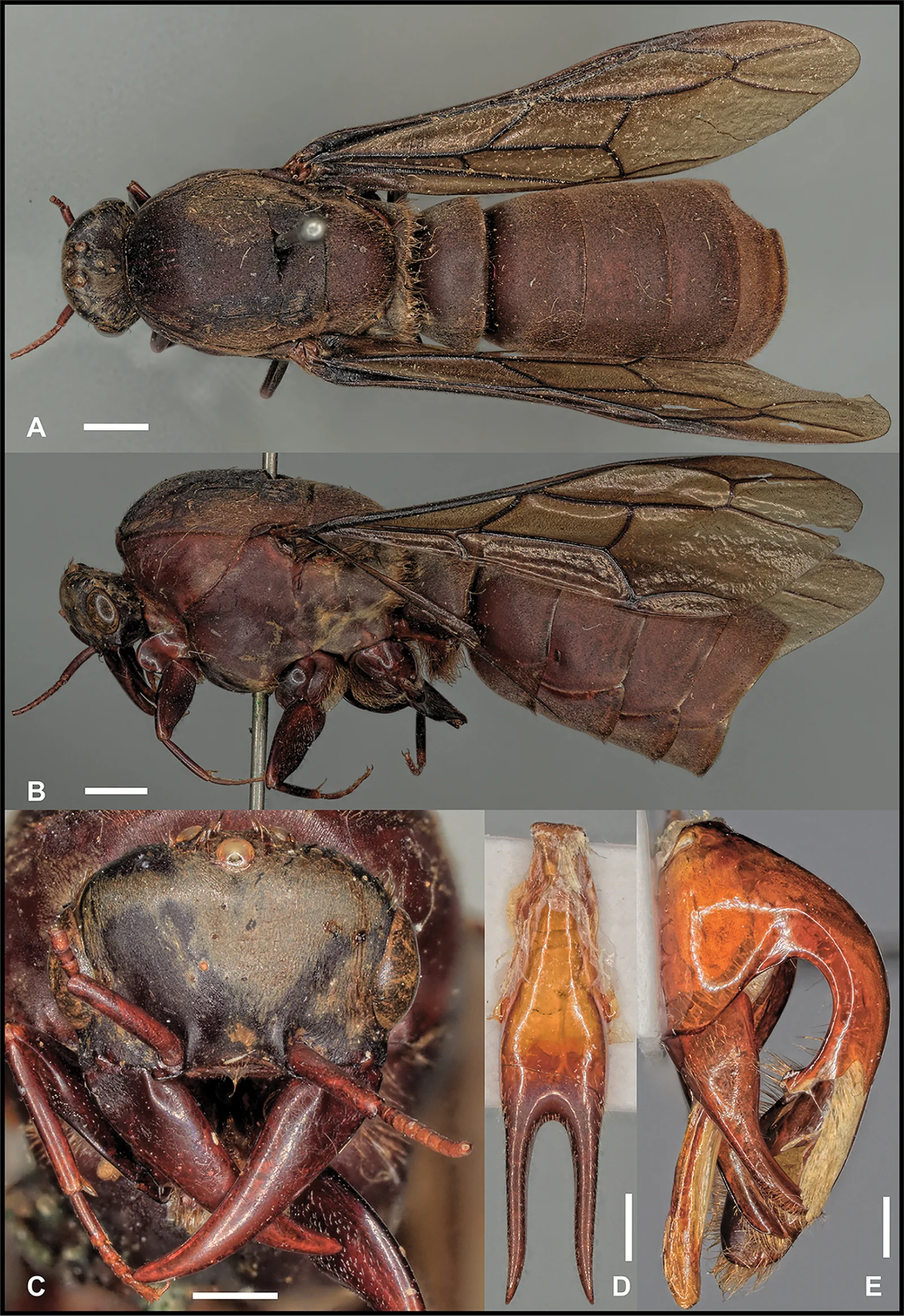
Syntype male specimen B of *Dorylus nigricans* in ZMHB. **A**. Dorsal overview; **B**. Lateral overview; **C**. Head in full-face view; **D**. Subgenital plate; **E**. Genital capsule. Scale bars: 2 mm (**A, B**); 1 mm (**C–E**). This specimen was designated as paralectotype.

**Habitus** (Fig. 11), larger than syntype B (Fig. 13) and similar to a freshly collected driver ant male from Taï National Park (CASENT 0172641, Figs 15, 16). **Head**: HW 5.331 mm, HL 3.317 mm, SL 1.972 mm, EL 1.971 mm, both antennae damaged, last flagellomeres missing; right antenna with 12 segments; scapus and 1^st^ and 2^nd^ funicles reddish-brown, colour of other segments not discernible due to dirt; antennal fossae pronounced, projecting over facial margin and transitioning into a prominent “tooth”; a second larger “tooth” – separated by a notch – on the medial side of the fossa; surface sculpture of head not discernible; mandibles slender and gently curved, anterolateral surface near base slightly concave; end of apical tooth rounded, not acute; clypeus covered with very long hairs projecting anteriorly between antennae; labrum covers mouth; palpi not visible. **Mesosoma**: MW 6.674 mm, HFL 4.117 mm, HTL 3.114 mm, CL 5.212 mm, surface in dorsal view covered with short adpressed yellow hairs; long hairs on coxa, ventral margin of mesopleuron and posterior margin of propodeum; wings translucent, veins black, forewing with anterior lateral margin yellow below costa. **Petiole**: PeW 5.742 mm, in dorsal view bowl-shaped; long hairs on ventral side of petiole; in dorsal view petiole covered with short adpressed yellow hairs as on the mesosoma. **Gaster**: PL 2.949 mm, PD 1.376 mm, SPWB 1.599 mm, in dorsal view covered with adpressed short yellow hairs; prongs of subgenital plate parallel. **Labels**: see Fig. 12.

#### Syntype B

**Figure 14.**
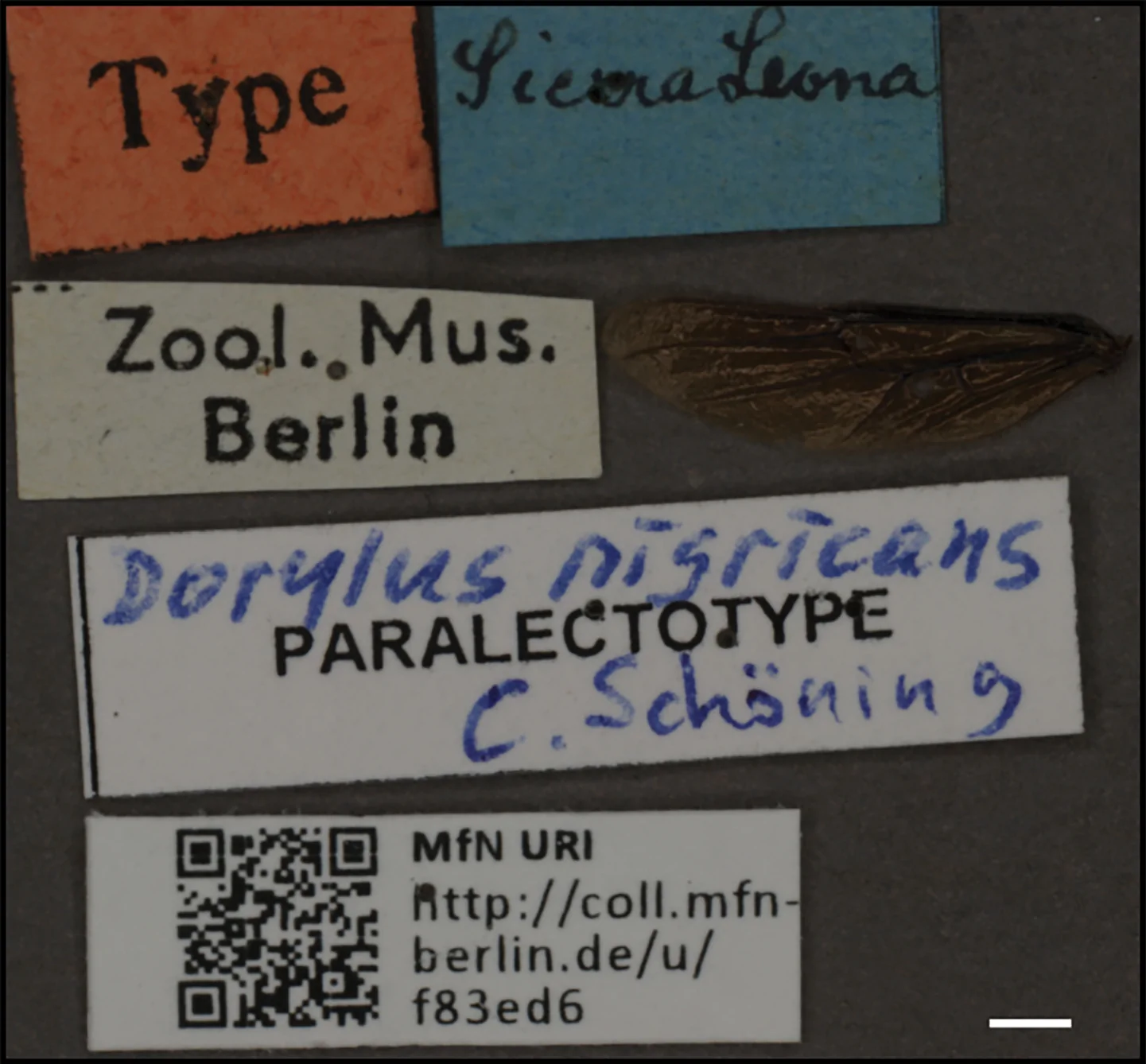
Labels of *Dorylus nigricans* syntype specimen B (Fig. 13) in ZMHB, designated as paralectotype. Scale bar: 2 mm.

**Habitus** (Fig. 13), considerably smaller than syntype A. **Head**: HW 4.379 mm, HL 2.852 mm, SL 1.776 mm, EL 1.516 mm, both antennae damaged, distal flagellomeres missing, colour of antennae reddish-brown; antennal fossae similar to those in syntype A, but slightly more pronounced; head apparently darker than in syntype A, might be an artifact; mandibles slender and gently curved, anterolateral surface near base concave, slightly stronger than in syntype A; end of apical tooth rounded, not acute; clypeus covered with very long hairs projecting anteriorly between the antennae; labrum covers mouth; palpi not visible. **Mesosoma**: MW 5.975 mm, HFL 3.665 mm, HTL 2.735 mm, CL 4.784 mm; in dorsal view covered with short adpressed yellow hairs; hairs less dense than in syntype A; propodeum damaged on right side; wings opaque and smoky brown over whole surface, posterior areas slightly brighter. **Petiole**: PeW 4.855 mm, in dorsal view bowl-shaped; long hairs on ventral side of petiole; in dorsal view petiole covered with short adpressed yellow hairs as on the mesosoma. **Gaster**: PL 2.737 mm, PD 1.339 mm, SPWB 1.469 mm, in dorsal view covered with adpressed short yellow hairs. **Labels**: see Fig. 14.

**Figure 15.**
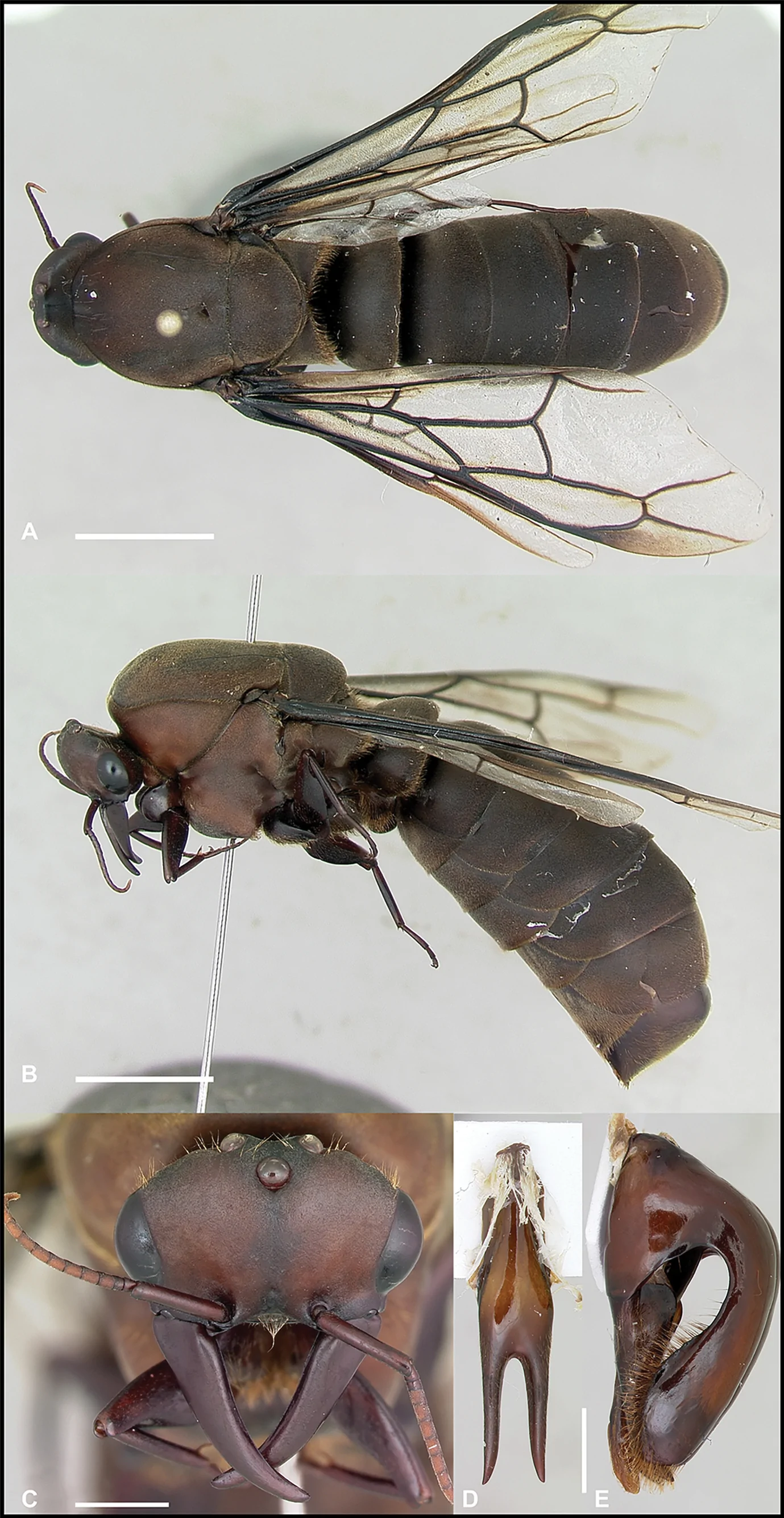
*Driver ant* male from Taï National Park, Ivory Coast, similar to syntype specimen A of *D. nigricans*. **A**. Dorsal overview; **B**. Lateral overview; **C**. Head in full-face view; **D**. Subgenital plate; **E**. Genital capsule. From AntWeb (CASENT0172641), photographs by April Nobile. Scale bars: 5 mm (**A, B**); 2 mm (**C–E**), with the last two sharing one scale bar.

**Figure 16.**
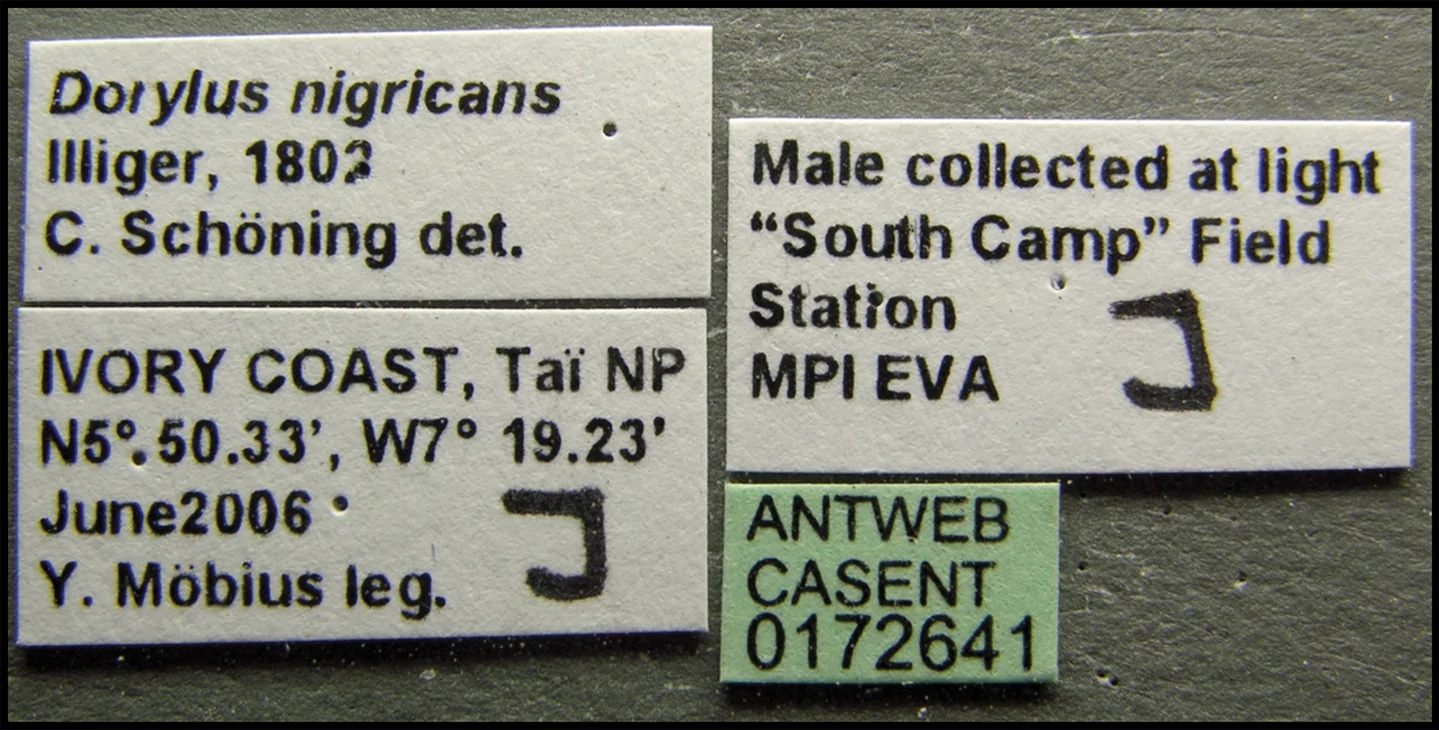
Labels of male specimen CASENT0172641 (Fig. 15).

**Figure 17.**
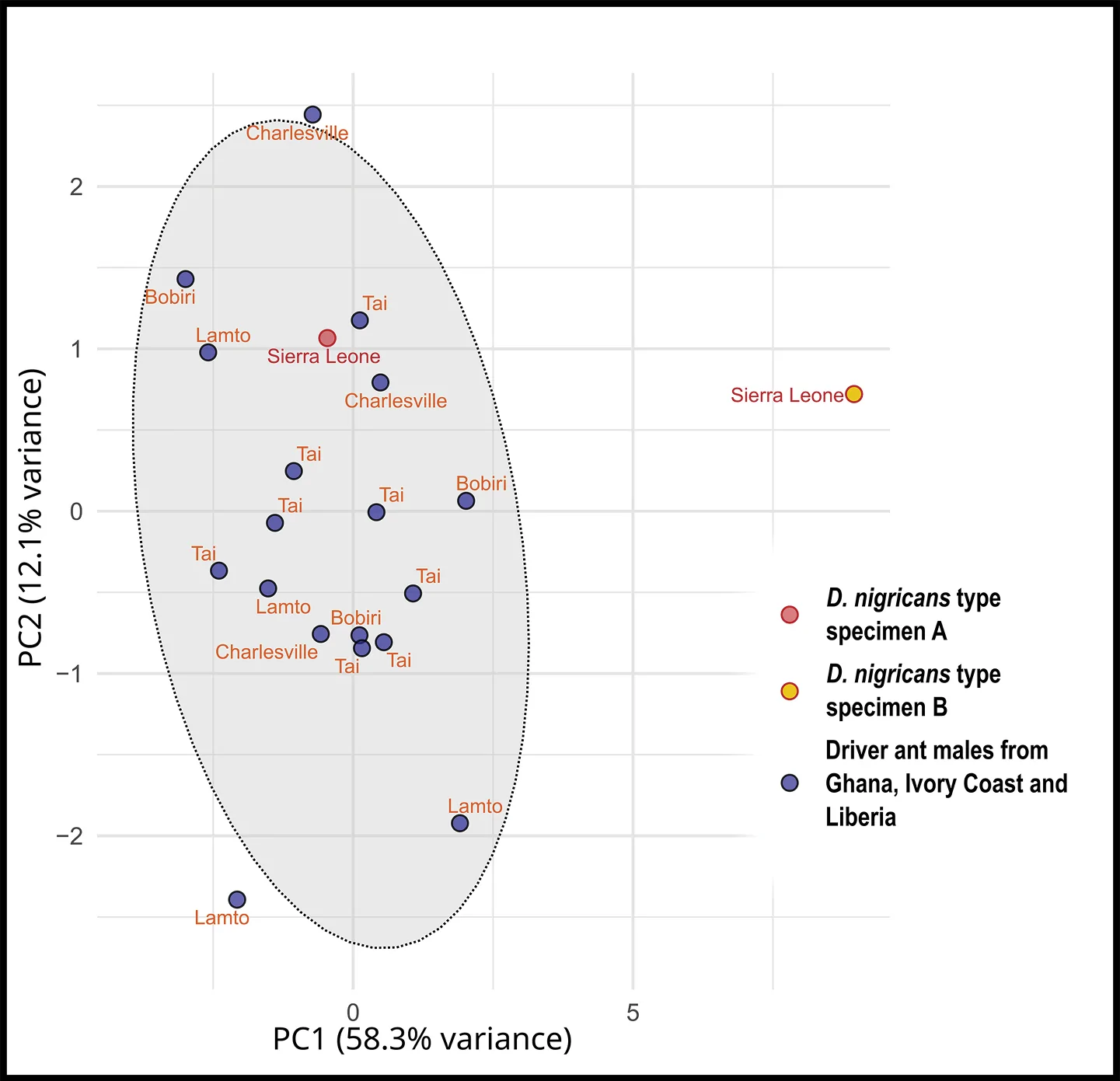
PCA based on ten scaled morphometric measurements of West African driver ant males (*n* = 18) in relation to syntype specimen A and syntype specimen B. The measurements are HW, SL, HL, EL, MW, PeW, HTL, CL, PL, PD, and SPWB.

Despite the unfavourable condition of the type specimens, it is obvious that they differ in size and wing colour. A morphometric comparison of the two type specimens with the driver ant males with translucent wings collected at Charlesville (Liberia), Bobiri (Ghana), Lamto and Taï (both Ivory Coast, one specimen depicted in Figs 15, 16) strongly suggests that syntype A is conspecific with them, while the substantially smaller syntype B belongs to a different species (Fig. 17).

### Matching the *D. nigricans* male to the corresponding workers

**Figure 18.**
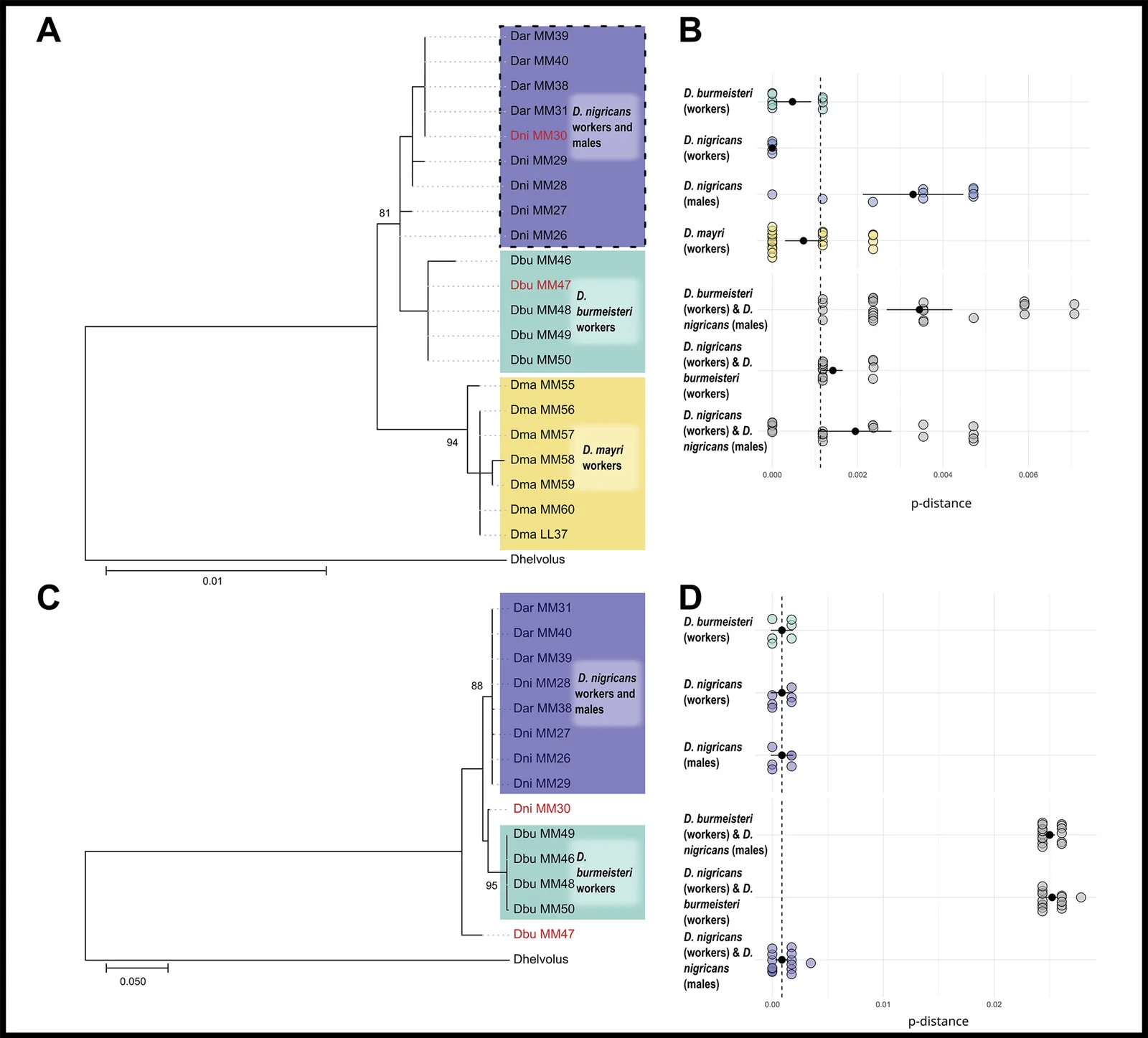
**A**. Maximum Likelihood (ML) dendrogram based on nuclear DNA sequences. The tree with the highest log likelihood is shown, including bootstrap values (1000 replicates) for well supported nodes (≥80). Coloured boxes delineate morphologically distinguishable taxa. Blue: *Dorylus* males and workers; yellow: *D. mayri*; green: *D. burmeisteri*. The box with a dashed frame highlights the *D. nigricans* group. The two samples shown in red font were outliers in the analysis of mitochondrial data (see **C, D**); **B**. Pairwise distances among nuclear DNA sequences. Colour codes as in **A**. The dashed line indicates the mean of all within species distances. Black points represent group mean distances, with 95% confidence intervals shown as horizontal black bars; **C**. ML dendrogram based on mitochondrial DNA sequences. Labels are the same as in **A**. Samples of *D. mayri* only produced pseudogene sequences and were therefore excluded from the analyses of mitochondrial data; **D**. Same analysis and labels as in **B**., but based on mitochondrial DNA sequences. Two sequences that were outliers in this analysis (shown in red in **C**) are not plotted here (see main text for details). Scale bars: indicate substitutions per site (**A, C**). *Dorylus helvolus* serves as the outgroup in **A, C**.

Based on nuclear DNA sequences (Table 2), the *D. mayri* workers are only distantly related to the two other worker-based species and the *D. nigricans* males (Fig. 18A). We were unable to sequence mitochondrial DNA from *D. mayri* specimens. Instead, all *D. mayri* specimens yielded a degenerated pseudogene instead, while the remaining samples produced regular mitochondrial sequences with the same PCR primers. The consistent amplification of a pseudogene in *D. mayri*, but not in any other samples, can be interpreted as a molecular character, consistent with the results from nuclear DNA sequences showing that *D. mayri* is genetically distinct and, therefore, does not correspond to the *D. nigricans* males.

For the examined nuclear DNA loci, the *D. nigricans* males were most closely related to workers corresponding to those originally described under the name *Anomma arcens*, and one of the males was genetically identical to some of these workers (Fig. 18A). Workers of *D. burmeisteri*, on the other hand, were more distantly related (Fig. 18A). The same pattern was apparent when comparing genetic distances directly (Fig. 18B). At the mitochondrial locus, *D. nigricans* shared a haplotype with workers corresponding to those originally described under the name *A. arcens*, while the *D. burmeisteri* haplotypes were more distantly related (Fig. 18C, D). One *D. nigricans* male (Dni MM30) and one *D. burmeisteri* worker (Dbu MM47) yielded mitochondrial haplotypes that did not cluster with the remaining haplotypes from the respective species (highlighted in red in Fig. 18C). However, the phylogenetic placements of these sequences were ambiguous, as evidenced by low bootstrap support values (Fig. 18C). It therefore remains unclear whether the three driver ant species at Taï National Park are reciprocally monophyletic at mitochondrial haplotypes. Paraphyly, if confirmed, would be reminiscent of patterns in East African driver ants (Kronauer et al. 2011) and might arise from hybridisation and mitochondrial introgression, incomplete lineage sorting, recent migration, or a combination of these and other factors. Paraphyletic mtDNA haplotypes within a species are a recurring phenomenon in ants and do not necessarily reflect recent gene flow (Seifert 2024). While the available data do not allow us to distinguish between the various possible scenarios, the two outliers neither affect the proposed male-worker association nor our conclusions with respect to species delimitation.

Taken together, our analyses of DNA sequence data from males and the three worker forms found at Taï National Park demonstrate that males similar to the *D. nigricans* syntype A are conspecific with workers originally described under the name *Anomma arcens* Westwood, 1847. Below we provide descriptions of all the known forms of the three species and carry out the necessary taxonomic changes.

### Taxonomic treatment of the three species

#### Dorylus burmeisteri

Taxon classificationAnimaliaHymenopteraFormicidae

(Shuckard, 1840)

4EB49FF7-8A39-579B-8606-1FE3511122C7

**Figure 19.**
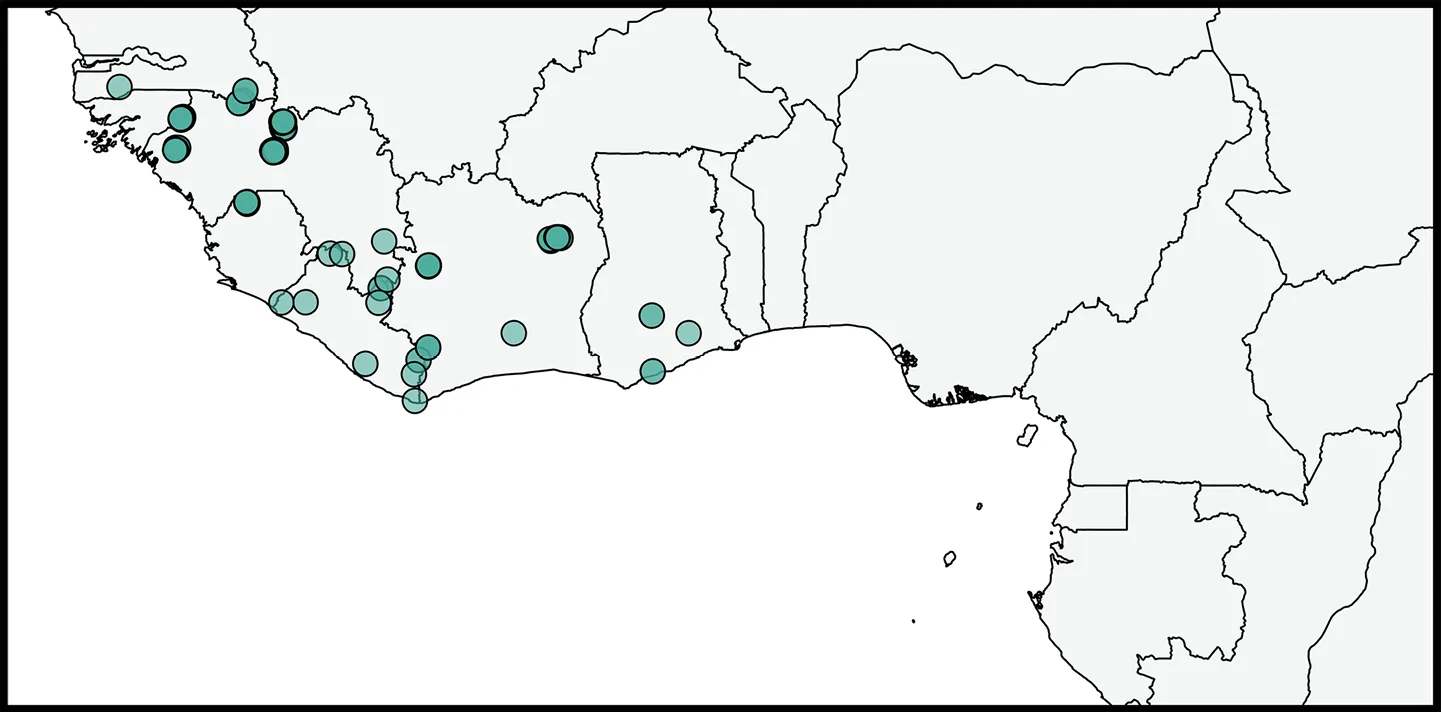
Distribution of *Dorylus burmeisteri*, with each dot representing a collection event.

**Figure 20.**
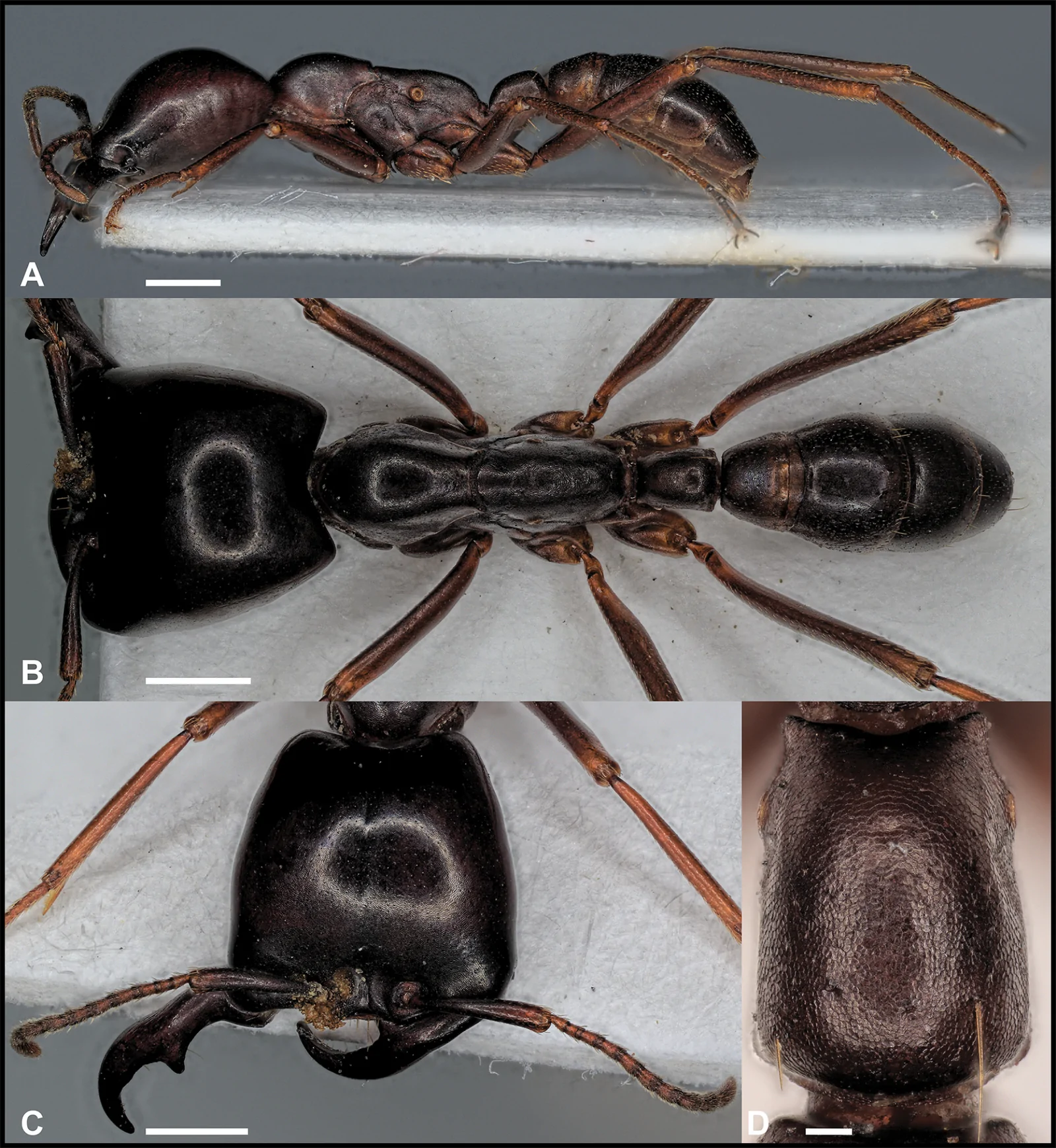
Neotype of *Anomma burmeisteri* (SMNG-SIE-2018-192-01) from Outamba. **A**. Lateral view; **B**. Dorsal view; **C**. Full-face view; **D**. Petiole in dorsal view. Scale bars: 1 mm (**A–C**); 0.1 mm (**D**).

**Figure 21.**
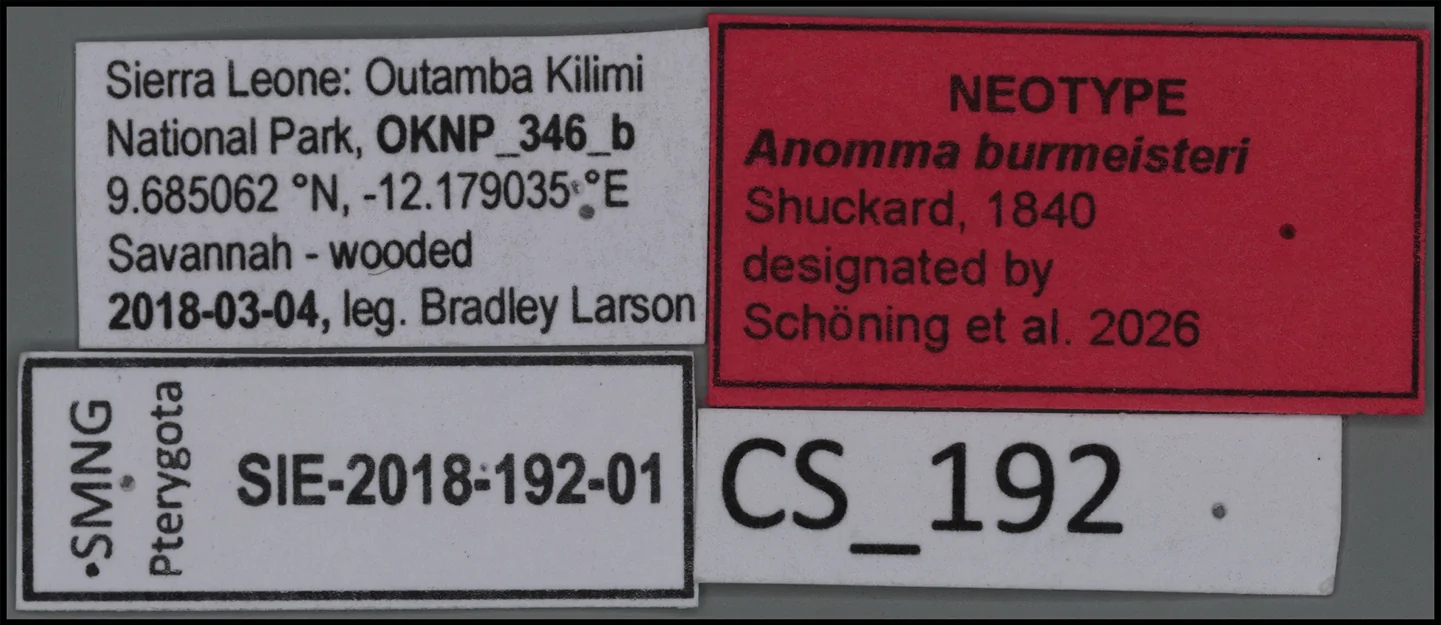
Labels of specimen SMNG-SIE-2018-192-01 (Fig. 20), designated as neotype of *Anomma burmeisteri* Shuckard, 1840. The current valid name is *Dorylus burmeisteri*.

**Figure 22.**
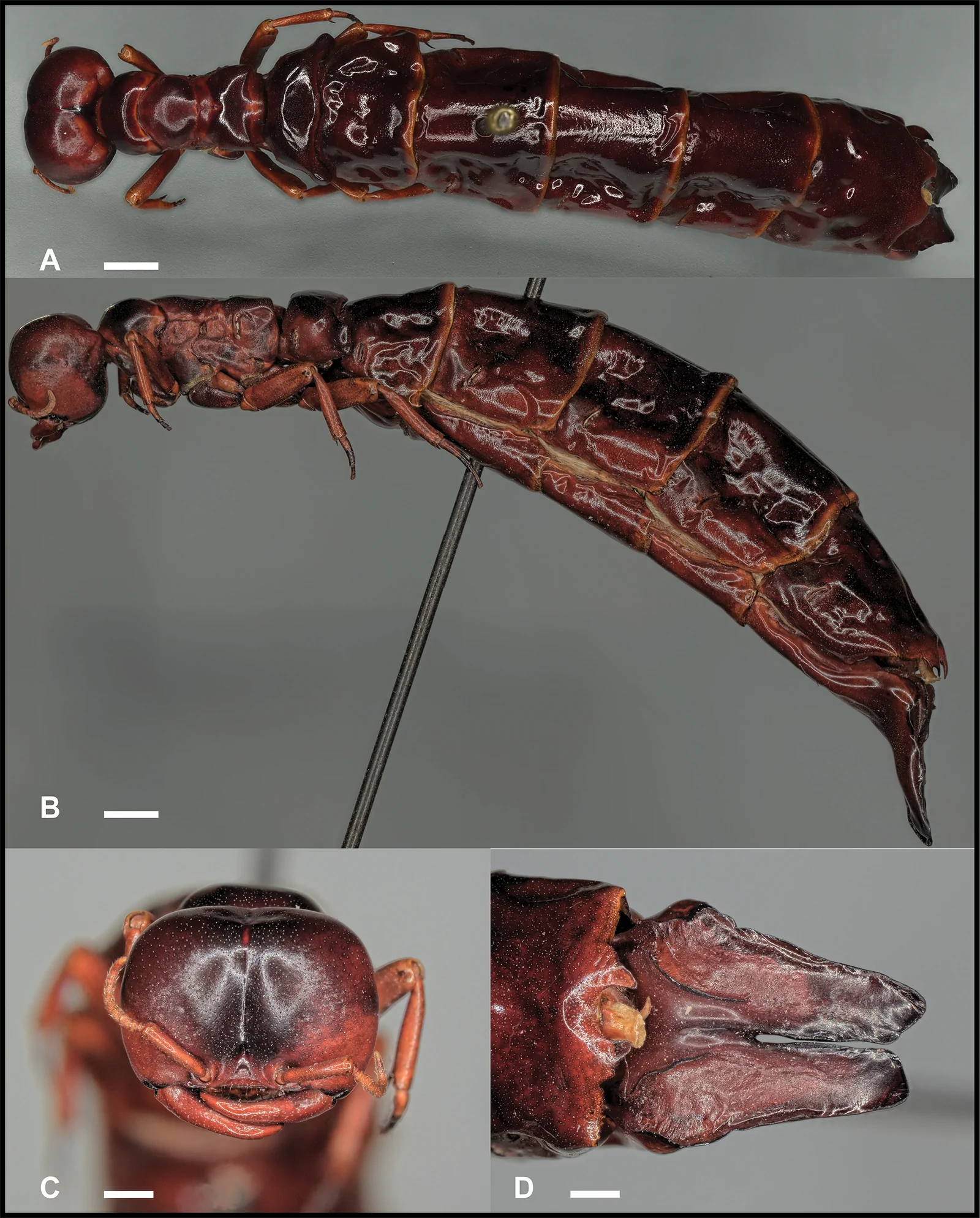
*Dorylus burmeisteri* queen (Van Boven 2767). **A**. Dorsal view; **B**. Lateral view; **C**. Full-face view; **D**. Dorsal view of hypopygium. Scale bars: 2 mm (**A, B**); 1 mm (**C, D**).

Figs 6D–F, 7B, 19–22

Anomma burmeisteri Shuckard, 1840: 326. Holotype worker, Sierra Leone (D.F. Morgan) [not examined, lost]. Neotype worker, here designated, Sierra Leone, Outamba Kilimi National Park, March 4^th^, 2018, Bradley Larson OKNP_346_b, SMNG-SIE-2018-192-01, SMNG.
*Dorylus* (*Anomma*) *nigricans* st. *burmeisteri* var. *hybrida* Santschi, 1912: 161. Four workers, Senegal: Casamance, collected by M. Claveau, NHMB. Infrasubspecific name unavailable according to Bolton (2026).

##### Annotation.

A comprehensive taxonomic history of this species can be found in AntCat.

##### Other material examined.

Ghana: • Bobiri (Lat. 6.68, Long. -1.35, 4 worker samples, L. Fisch & W.H. Gotwald Jr., CSAC), • Efufu (Lat. 5.20, Long. -1.32, 2 worker samples, L. Fisch & W.H. Gotwald Jr., CSAC), • New Tafo (Lat. 6.22, Long. -0.37, 1 worker sample, L. Fisch & W.H. Gotwald Jr., CSAC). Guinea: • Bakoun (Lat. 11.67, Long. -11.8, 12 worker samples, A. Agbor, SMNG), • Bossou (Lat. 8.65, Long. -11.8, > 30 worker samples, T. Humle, K. Koops, Y. Sugiyama, CSAC), • Sangaredi (Lat. 11.1, Long. -14.05, 10 worker samples, A. Keita, B.A. Sefou, C. Kabinet, SMNG), • Seringbara (Lat. 7.64, Long. -8.41, several hundred worker samples, K. Koops, CSAC), • Sobeya (Lat. 11.0, Long. -11.43, 64 worker samples, B. A. Sefou, G. Brazzola, SMNG), • Sobory (Lat. 11.82, Long. -11.21, 28 worker samples, A. Toure, A. Agbor, SMNG), Guinea-Bissau: • Boe (Lat. 11.93, Long. -13.90, 46 worker samples, E. Ton & J. van Schijndel, SMNG), Ivory Coast: • Comoé (Lat. 8.76, Long. -3.85, 26 worker samples, J. Beck, C. Kost, P. Kouamé & J. Lapuente, CSAC, SMNG; Kronauer et al. 2007/5, NHMD), • Gestion Participative des Ressources Naturelles et de la Faune (Lat. 8.71, Long. -4.05, 20 worker samples, A. Tickle, SMNG), • Lamto (Lat. 6.22, Long. -5.03, 8 worker samples, 1 worker sample with queen, Y. Kolo, J.M. Leroux, J. Levieux, J.K.A. van Boven, CASC, MCZC, RMCA), • Mont Sângbé, (Lat. 8.00, Long. -7.31, 22 worker samples, H. Cohen & J. Preece, SMNG), • Taï National Park (Lat. 5.84, Long. -7.32, > 20 worker samples, T. Deschner, J. Lapuente, L. Luncz, A. Meier, Y. Möbius, C. Schöning, CASC, SMNG). Liberia: • East Nimba (Lat. 7.43, Long. -8.57, 14 worker samples, S. Jones & S. Maroccoli, SMNG), • Grebo (Lat. 5.48, Long. -7.56, 2 worker samples, S. Marrocoli, SMNG), eight other sites in Liberia (from nationwide survey, see Tweh et al. 2014). Senegal: • Dindefelo (Lat. 12.37, Long. -12.31, 21 worker samples, P. Álvarez, A. Aviles, A. Barciela, G. Coppola, J.M. Garcia, M. Llana, V. Moreno, SMNG), • Fongoli (12.65, Long. -12.20, 11 worker samples, S. Bogart, W.C. McGrew, J. Pruetz, CSAC), Sierra Leone: • Outamba Kilimi National Park (Lat. 9.68, Long. -12.16, 27 worker samples, B. Larson, SMNG).

##### Notes.

The species was originally described based on a single worker collected in Sierra Leone. Unfortunately, the type specimen has been lost. In the species description, Shuckard (1840) emphasised features of the worker that distinguish the newly erected genus *Anomma* from the genus *Typhlopone* described by Westwood (1839). The general description is therefore applicable to almost all driver ant species and lacks details that would be helpful in separating this species from other driver ant species. However, Shuckard additionally wrote “Brightly shining, perfectly smooth, pitchy black, with the antennae, legs and thorax, ventral incisures and sides of the abdomen pitchy red”. In the workers collected in the present study, the head surface is indeed shiny and lacks the fine reticulation found in *D. nigricans* workers. Interestingly, Smith (1858) examined the type specimens of both *Anomma burmeisteri* Shuckard, 1840 and *Anomma arcens* Westwood, 1847. His account is somewhat confusing because, when he uses the words “former” and “latter”, it is unclear whether he refers to the sequence of the two species names in his species list or in his introductory sentence in which the sequence is reversed compared to the list. However, when comparing the worker morphology of the two species he likewise stressed the head surface sculpture as a diagnostic feature: “highly polished, perfectly smooth, entirely destitute of punctures or sculpture” (*A. burmeisteri*) vs “subopake [sic!] and covered with a very delicate reticulation” (*A. arcens*). Moreover, he mentions the lateral angles of the head being more rounded in *Anomma burmeisteri*. While such features vary with size in driver ants, we assume that Smith was referring to large individuals with only one subapical tooth as they are depicted in his head shape drawings (Smith 1858: pl. VIII, figs 2, 3). In such larger workers the differences clearly exist. Finally, in these drawings the *Anomma burmeisteri* worker has a shorter scape relative to its head width (1.5 units / 3.2 units ≈ 0.47) than the *Anomma arcens* worker (1.5 units / 2.85 units ≈ 0.53), as we also find in our present morphometric analyses (Fig. 9). Together, this leads us to the conclusion that the workers are conspecific with the worker specimen described as *Anomma burmeisteri* by Shuckard. During a visit of the Hymenoptera collection of the Natural History Museum in London by one of us (CS) in 2005 the type specimen could not be located. An additional inquiry confirmed that the specimen is lost (Suzanne Ryder, pers. comm. 11 February 2026). The loss of the type specimen necessitates the designation of a neotype in order to clarify the taxonomic status. We chose a specimen collected from Sierra Leone, the same country where the original type specimen had been collected.

##### Description of the neotype

**(by current designation)**. Worker specimen SMNG-SIE-2018-192-01 chosen from sample OKNP_346_b (collected by Bradley Larson on 4 March 2018 at Outamba Kilimi National Park in Sierra Leone). HW 2.620 mm, SL 1.364 mm, PW 1.179 mm, HFL 2.830 mm, HTL 2.773 mm, PeW 0.607 mm, PeL 0.812 mm. Head surface shiny and lacking the fine reticulation found in *D. nigricans*, typical trapezoidal head shape of large driver ant workers (anteriorly wider than posteriorly). ***Head***: Fig. 20C, dark brown with black patches in median line and at the anterior as well as posterior rim. Antenna with eleven antennomeres. Scape dark brown, but lighter at distal end; surface shinier and with weaker microsculpture than in similarly-sized workers of *D. nigricans*. Head trapezoidal, anteriorly wider than posteriorly. Mandible with one subapical tooth only, claw-like shaped, with strongest curvature at distal fourth. ***Mesosoma***: Fig. 20A, B. Pronotum dark brown to red brown with lighter areas in vicinity of sutures. Black line runs parallel to posterior rim of pronotum. Propodeum dark brown and with similar appearance as pronotum. Propodeal spiracle surrounded by a pale brown, yellowish ring, separated from the dark brown surroundings by a slim black ring. Coxae red brown, and mesocoxae and metacoxae with paler brown pigmentation distally and in lateral groove. Femora brown, yellow brown at joints. Tibiae appear paler than femur, with only slightly darkened mid-section. Tarsi similar in colour to tibiae. Two claws on each tarsus yellow proximally, black distally. Prothoracic leg darker than the other two legs. ***Petiole***: Fig. 20D. In dorsal view, posterior “tubercles” visible and producing widest part of petiole; colouration similar to mesonotum with dorsal surface darker than ventral one. ***Gaster***: Fig. 20A, B. First tergite dark brown with pale transverse band posteriorly. Gaster tergites one to four with lighter area close to the lateral margins, widening posteriorly and partly following their posterior end. ***Labels***: Fig. 21.

##### Description of the queen.

This description is based on one queen associated with workers collected from a single nest at Lamto in Ivory Coast (Van Boven and Levieux 1970) and obtained from the Van Boven collection in the RMCA (collection number Van Boven 2767). ***Habitus***: Fig. 22, smaller and brighter in appearance than *D. nigricans* (see Fig. 25). ***Total length***: 3.700 cm. ***Head***: Fig. 22C, HW = 5.384 mm, HL = 3.701 mm, SL = 1.734 mm, lateral sides red, dark brown on dorsal and ventral side, surface appears slightly dull due to microreticulation but weaker than in *D. nigricans*, foveola evenly distributed over whole surface of head and at least as deep as wide (diameter 27 µm), foveola occasionally with small hair-like sensillae (7 µm length), hairs usually pointing inwards to the centre of the foveola, no pilosity present; ***Antenna***: 11 antennomeres, funicular segments dark orange and scape slightly darker, antennal fossae pronounced, deeply impressed and rounded, median line V-shaped impressed in the lower half, clypeus with some setae of two different lengths, labrum hardly visible; ***Mandible***: some setae at inner side; no subapical teeth, apical tooth is slightly pointing medially; posterior angles of head drawn out backwards and ventrally. ***Mesosoma***: Fig. 22A, B, PW = 3.309 mm, PropW = 3.485 mm, HFL = 3.435 mm, colour dark brown like dorsal areas on head, paler close to sutures between segments, promesonotal suture slightly impressed, ventral margin of pronotum with small setose ridge. ***Propodeum***: wider than pronotum, not rectilinear, lateral margins curved, left posterior margin with small pronounced spine, propodeal spiracles visible in dorsal view, at metanotal groove with medial spine projecting backwards, surface morphology similar to head, with microreticulation and foveolae, coxae with yellowish pilosity at junctions, terminal tarsal segments missing from specimen. ***Petiole***: Fig. 22A, B, PeW = 5.855 mm, colour similar to the darker areas of the mesosoma, surface sculpture similar to head and mesosoma but microreticulation not as strong in medial areas, no seta and without pilosity, posterior lateral lobes very conspicuous in dorsal view. ***Gaster***: Fig. 22A, B blackish brown, shinier than head and mesosoma but otherwise similar, numerous scratches of unknown origin. ***Genitalia***: Fig. 22D, typical driver ant hypopygium similar as depicted in Gotwald (1982: 167).

##### Biology.

Confirmed records treated here extend from Senegal to Ghana; the status of related forms east of the Dahomey Gap requires further study. The species is not restricted to moist forests (where it often coexists with *D. nigricans* and *D. mayri*) but also occurs in drier savanna areas. It was observed in more detail during fieldwork at Taï National Park, where we observed nesting and swarm raiding behaviour typical for driver ants. The species is regularly consumed by chimpanzees at Bossou, Fongoli, Seringbara, and Taï (Schöning et al. 2008c; Koops et al. 2015).

##### Remarks.

Van Boven and Levieux (1970) conducted field studies at Lamto, Ivory Coast, during which they also excavated queens from nests. These queens are described here. Van Boven and Levieux (1970) mentioned the occurrence of “*Dorylus* (*Anomma*) *burmeisteri* var. *molestus* (Gerstäcker)” as well as of “*Dorylus* (*Anomma*) *nigricanssjöstedti* (sic!) Emery”. Leroux (1982) studied “*Anomma nigricans* Illiger” at Lamto for several years and reported extensive data on nesting, foraging and emigration behaviour as well as on reproduction. Examination of the queens and workers collected by Van Boven and Levieux (1970) and held in RMCA, and of the workers collected by Leroux and originally sent to W.H. Gotwald (now held in MCZC), revealed that the three authors in fact studied colonies of two different species, namely *D. burmeisteri* and *D. nigricans*. The colonies at Lamto designated by Leroux (1982) as MS, ESB, IS, EY, 27G and D belong to *D. burmeisteri*.

The workers described as *Dorylus* (*Anomma*) *nigricans* st. *burmeisteri* var. *hybrida* Santschi, 1912 (unavailable name) do not show an aberrant morphology that would justify treating them as a separate species. We consider them conspecific with *Dorylus burmeisteri*.

The male of this species is unknown. Emery (1895) described a male from Ghana (no information on the specific locality) as a new variety (“*funereus*”) and mentions the distinctly darker wings as a relevant feature in which it differs from *D. nigricans* males. We have not been able to examine this specimen in detail. Using the images available on AntWeb (specimen CASENT0903695, https://www.antweb.org/specimen.do?code=casent0903695) we estimate its head width to be 5.22 mm, which is much larger than the head width of the smaller *D. nigricans* type specimen B (see below). This *D. funereus* male might therefore belong to *D. burmeisteri*, which indeed occurs in Ghana.

#### Dorylus mayri

Taxon classificationAnimaliaHymenopteraFormicidae

Santschi, 1912

6D81ECC5-01E8-566E-9C07-7F015B0DAAB0

Figs 6G–I, 7C, 23

Dorylus (Anomma) mayri Santschi, 1912: 159. Three worker syntype specimens, Cameroon, locality and collector unknown (these were sent by Emery, who had received them from Mayr), NHMB [examined].

##### Other material examined.

Cameroon: • Dja (Lat. 3.62, Long. 13.68, 5 worker samples, I. Deblauwe, CSAC), • Korup (Lat. 5.35, Long. 9.04, 7 worker samples, J. Schäfer, CSAC), • Kom Wum (Lat. 6.42, Long. 10.26, 14 worker samples. C. Fotang, CSAC), Central African Republic: • Bai Hokou (Lat. 2.86, Long. 16.47, 1 worker sample, C. Cipoletta, CSAC). Gabon: • Loango (Lat. -1.99, Long. 9.65, 4 worker samples, C. Boesch & J. Head, CSAC), • Monts de Cristal (Lat. 0.82, Long. 10.16), 1 worker sample, C. Schöning, CSAC), Ghana: • Kakum (Lat. 5.48, Long. -1.35, 1 worker sample, W.H. Gotwald Jr. & L. Fisch, CSAC). Guinea: • Bossou (Lat. 8.65, Long. -11.8, > 30 worker samples, T. Humle, K. Koops, Y. Sugiyama; Kronauer et al. 2007/6, NHMD), • Seringbara (Lat. 7.64, Long. -8.41, > 100 worker samples, K. Koops). Ivory Coast: • Taï National Park (Lat. 5.84, Long. -7.32, > 20 worker samples, T. Deschner, J. Lapuente, L. Luncz, A. Meier, Y. Möbius, C. Schöning, CASC, SMNG). Liberia: • East Nimba (Lat. 7.43, Long. -8.57, 14 worker samples, S. Maroccoli & S. Jones, SMNG), • Grebo (Lat. 5.48, Long. -7.56, 17 worker samples, S. Marrocoli, SMNG), eight other sites in Liberia (from nationwide survey, see Tweh et al. 2014). Nigeria: • Cross River National Park (Lat. 5.58, Long. 8.75, 3 worker samples, M. Moffett & C. Schöning, Republic of Congo: • Goualougo (Lat. 2.66, Long. 16.66, 9 worker samples, D. Morgan & C. Sanz, CSAC).

**Figure 23.**
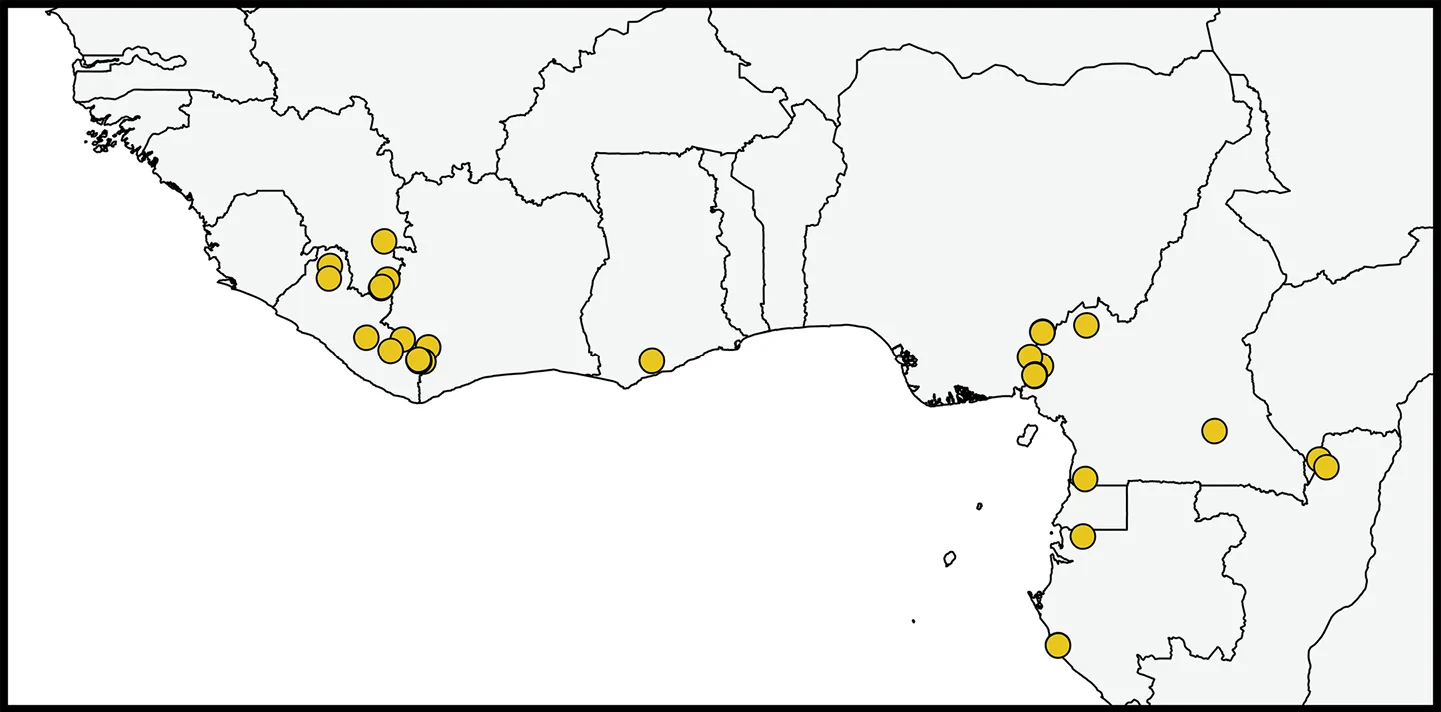
Distribution of *Dorylus mayri*, with each dot representing a collection event.

##### Biology.

In the new surveys, the species was found in moist forest habitats extending from Ghana to Liberia (Longitude -1.35 to -9.9). Other sampling efforts confirm its presence in areas eastwards towards Central Africa. The species was observed in more detail during fieldwork at Taï National Park, where we observed nesting and swarm raiding behaviour typical for driver ants. The species is regularly consumed by chimpanzees at Bossou, Dja, Goualougo, Seringbara and Taï (Schöning et al. 2008c; Koops et al. 2015; Sanz et al. 2010).

##### Remarks.

The male of this species is unknown. It is possible that it was described under another name but the crucial association between a male form and the name-bearing worker type specimen has not been established. Cohic (1948) described the mouthparts of a *D. mayri* queen, but no associated workers are depicted in the article so that the specimen’s identity could not be verified. Phylogenetic analyses based on mitochondrial and nuclear DNA sequence data support a sister relationship between *D. mayri* and all other driver ant species (Kronauer et al. 2007).

#### Dorylus nigricans

Taxon classificationAnimaliaHymenopteraFormicidae

Illiger, 1802

3E0A980B-F03D-5481-BC9A-B94C5B59D2AD

Figs 6A–C, 7A, 11, 12, 15, 16, 24, 25, 27

Dorylus nigricans Illiger, 1802: 188. Two male syntypes, Sierra Leone, collector unknown (“Hellwig’s collection”), ZMHB [examined]. Lectotype male (above referred to as syntype A), here designated, unique specimen identifier of lectotype: MfN 9e9e77.
Anomma pubescens Roger, 1861: 47. Holotype worker, Liberia, collector F.E. Guérin-Méneville (ZMHB) [examined]. Junior synonym of D. nigricans (see Emery 1895).
Combination in Dorylus (Anomma): Emery 1895: 710.
Combination in Anomma: Menozzi 1942: 165.
Combination in Dorylus: Borowiec 2016: 117.
Anomma arcens Westwood, 1847: 17. Thirty worker syntypes, Liberia, collected by Savage at Cape Palmas, OUMNH HYME1178/1-30 [examined]. New synonymy.

##### Annotation.

A comprehensive taxonomic history of this species can be found in AntCat.

**Figure 24.**
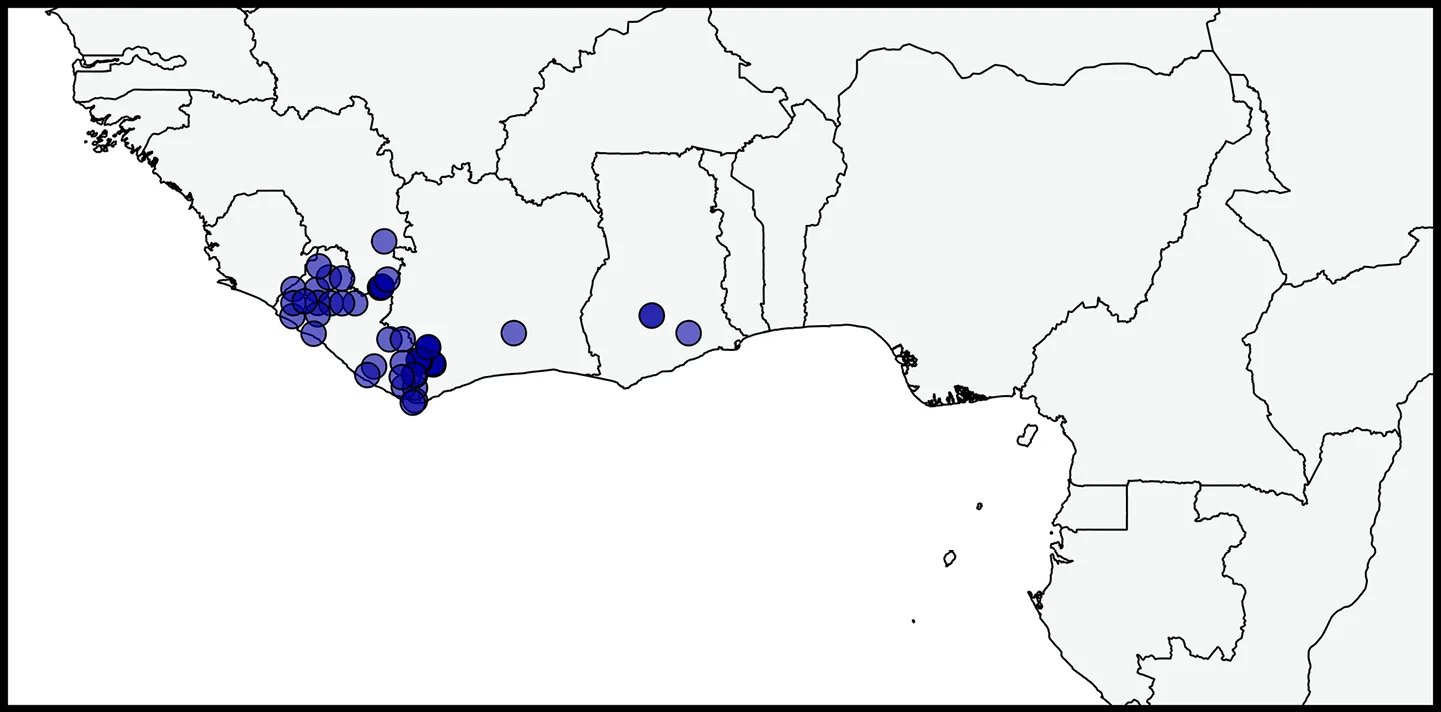
Distribution of *Dorylus nigricans*, with each dot representing a collection event.

**Figure 25.**
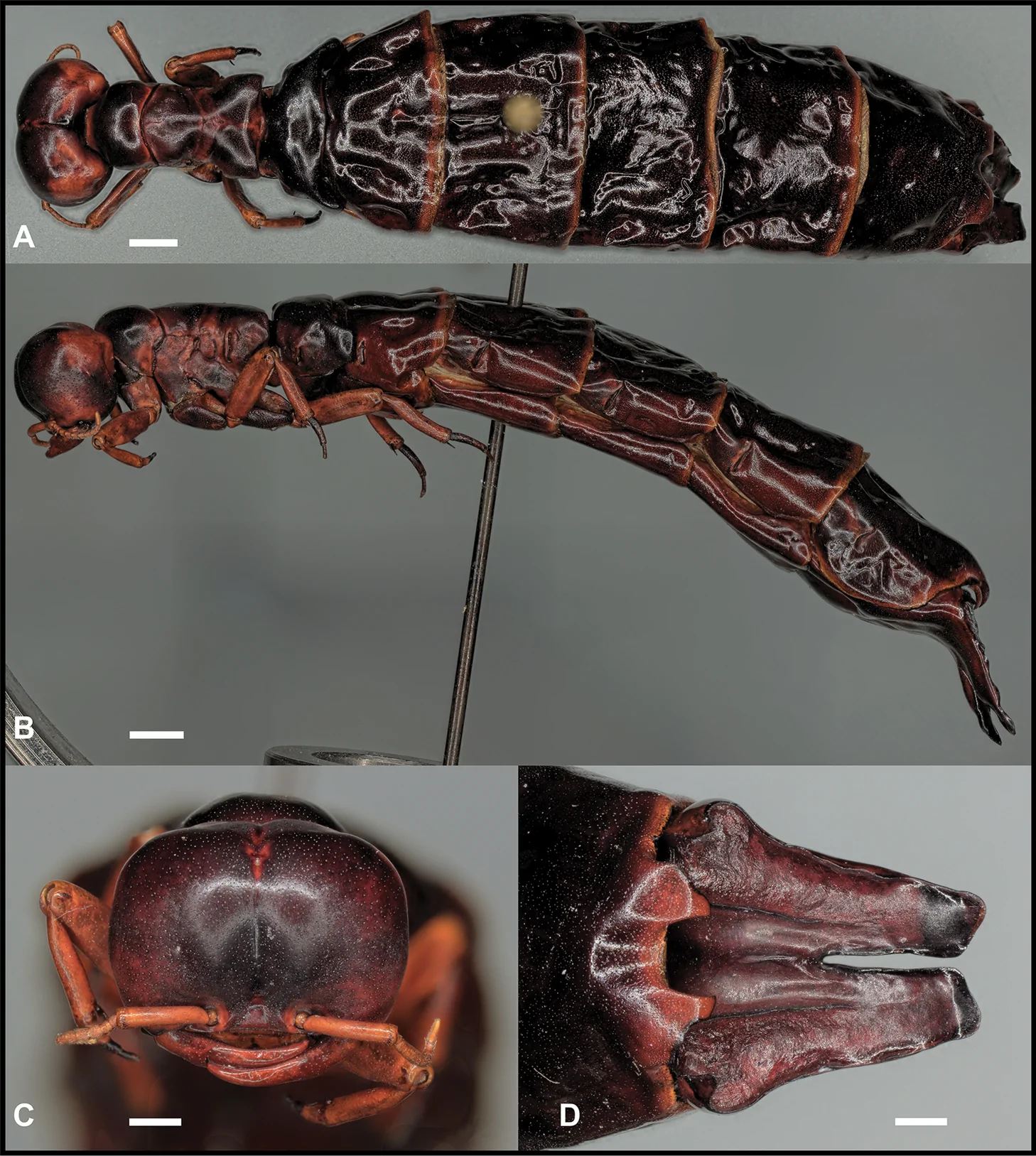
*Dorylus nigricans* queen (Van Boven 2768). **A**. Dorsal view; **B**. Lateral view; **C**. Full-face view; **D**. Dorsal view of hypopygium. Scale bars: 2 mm (**A, B**); 1 mm (**C, D**).

**Figure 26.**
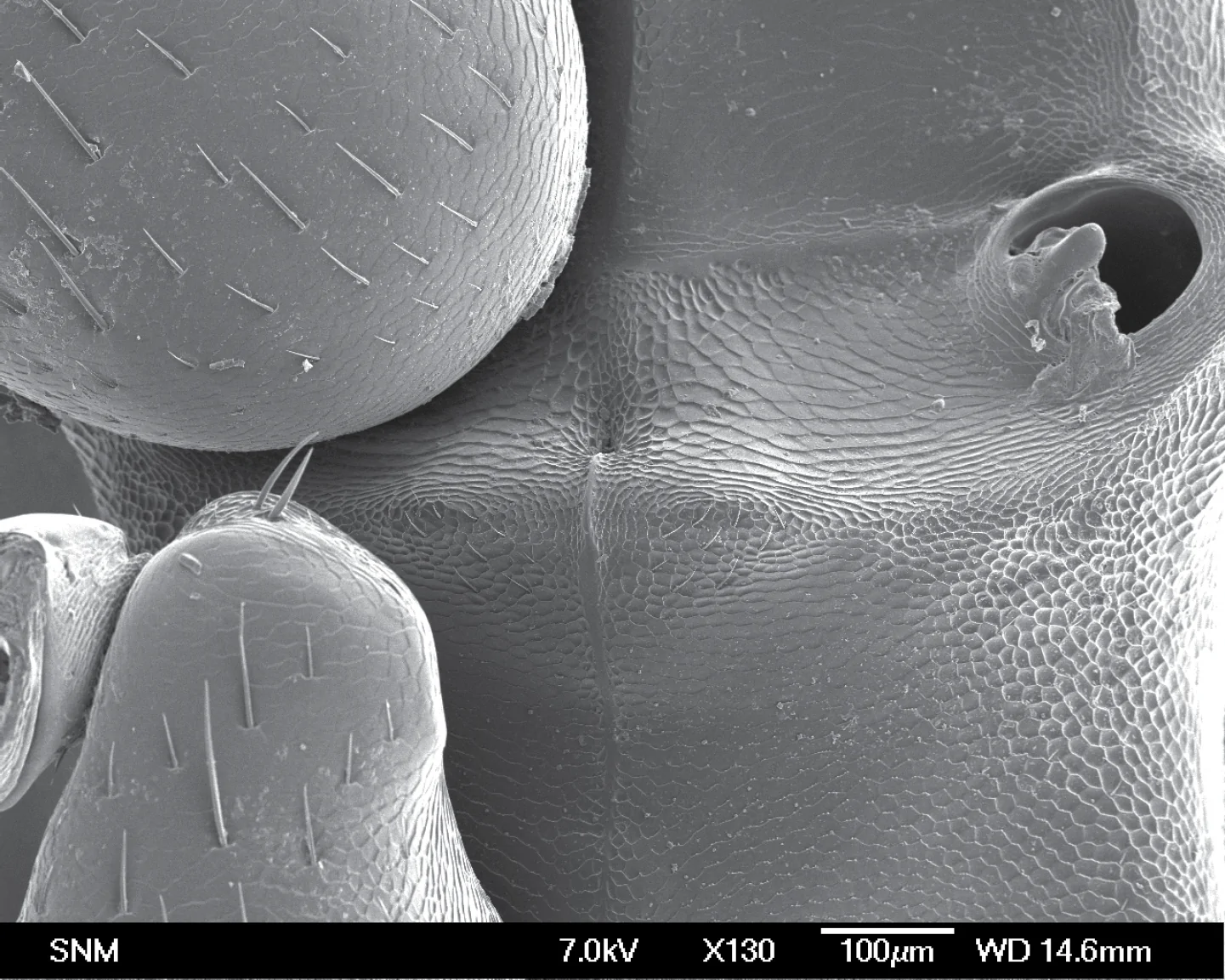
SEM picture of the basisternum of a large *Dorylus gribodoi* worker (HW 2.40 mm).

**Figure 27.**
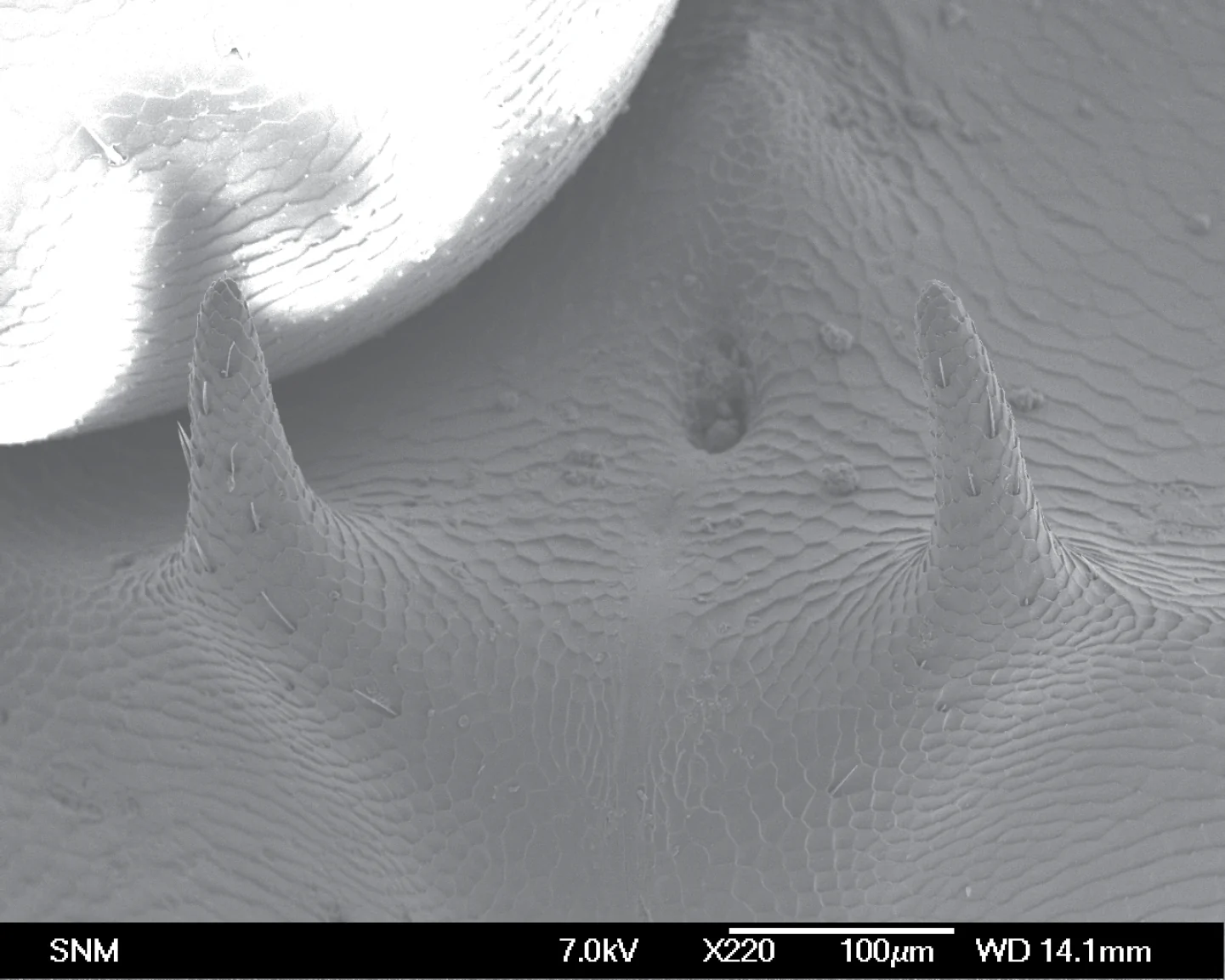
SEM picture of basisternal spines of a large *Dorylus nigricans* worker (HW 3.58 mm).

##### Other material examined.

Ghana: • Bobiri (Lat. 6.68, Long. -1.35, 3 males, 15 worker samples, L. Davis, L. Fisch, M. Moffett & W.H. Gotwald Jr., CSAC), • New Tafo (Lat. 6.22, Long. -0.37, 2 worker samples, L. Fisch & W.H. Gotwald Jr., CSAC). Guinea: • Bossou (Lat. 8.65, Long. -11.8, > 30 worker samples, T. Humle, K. Koops, Y. Sugiyama), • Seringbara (Lat. 7.64, Long. -8.41, > 100 worker samples, K. Koops). Ivory Coast: • Djouroutou (Lat. 5.40, Long. -7.20, 6 worker samples, J. Lapuente), • Lamto (Lat. 6.22, Long. -5.03, 2 males, 5 worker samples, 1 worker sample with queen, M. Kone, J.M. Leroux, J. Levieux, J.K.A. van Boven, CASC, MCZC, RMCA), • Taï National Park (Lat. 5.84, Long. -7.32, 10 males, > 20 worker samples, T. Deschner, J. Lapuente, F. Leendertz, L. Luncz, A. Meier, Y. Möbius, C. Schöning, CASC, SMNG; • 1 worker, Kronauer et al. 2007/7, 1 male, Kronauer et al. 2007/14, NHMD). Liberia: • Charlesville (Lat. 6.20, Long. -10.38, 3 males, D.H. Kistner, RMCA), • East Nimba (Lat. 7.43, Long. -8.57, 14 worker samples, S. Maroccoli & S. Jones, SMNG), • Grebo (Lat. 5.48, Long. -7.56, 54 worker samples, S. Marrocoli, SMNG), • 20 other sites in Liberia (from nationwide survey, see Tweh et al. 2014).

**Notes**. After the species was described based on the males collected in Sierra Leone, Savage (1849) discovered winged males in a driver ant colony in Gabon and considered them to belong to the same species. Since then, several authors have jumped to the conclusion that *Dorylus nigricans* males may be conspecific with driver ant forms collected at other locations across Africa. For example, Emery (1895) considered *Dorylus nigricans* males to be conspecific with *Anomma burmeisteri* workers, while Forel (1912) described a small queen from central Uganda under the name *D. nigricans*. Driver ant workers from other parts of Africa that resembled the West African *Anomma burmeisteri* were likewise described as subspecies of *D. nigricans* (e.g. “*Dorylus nigricans sjöstedti* (sic!)” by Emery (1899)). In all these instances the logic behind these decisions remains unconvincing because conspecificity can only be unequivocally demonstrated in one of the ways mentioned in the Introduction. The phrasing in Illiger’s original description from 1802 is somewhat ambiguous. We considered the possibility that he examined more than just two specimens and designated all of them as types, but no further specimens are present in the ZMHB collection. Bolton (2026) mentions that a part of Illiger’s collection is kept at the Staatliches Naturhistorisches Museum at Braunschweig (Germany), but this collection does not contain any further type specimens either (Claudia Kamcke, pers. comm. 27 August 2025), so that we could only work with the two specimens available.

The results presented above allow us to clarify the taxonomy of this species. We explain the argument behind synonymising *D. nigricans arcens* (Westwood, 1847) under *D. nigricans* Illiger, 1802 in a set of summary statements:

1. *Dorylus nigricans* Illiger, 1802 is a name based on a male from Sierra Leone.
2. Based on our morphometric analyses (Fig. 17) we consider the two male syntypes in ZMHB to belong to different species: syntype A falls within the variation of modern West African driver ant males from Charlesville (Liberia), Bobiri (Ghana), Lamto and Taï (Ivory Coast) with translucent wings, while the smaller syntype B with smoky-brown wings differs substantially from them. We thus conclude that syntype A is conspecific with the males with translucent wings.
3. We designate syntype A as the lectotype of *Dorylus nigricans* Illiger, 1802, thereby fixing the name to a single name-bearing specimen for the first time. Syntype B becomes the paralectotype and loses name-bearing function for *D. nigricans*; its species identity remains to be resolved.
4. The DNA sequence data from sympatric specimens from Taï National Park (Fig. 18) show that the males which we consider to be conspecific with syntype A are associated with the worker form with relatively longer legs and antennae.
5. The morphological examination of the worker syntypes of *Anomma arcens* Westwood, 1847 in OUMNH revealed that the workers with relatively longer legs and antennae are conspecific with those syntypes.
6. When combining steps 1–5, we conclude that the worker form historically called *A. arcens* is conspecific with the male-based *D. nigricans*. Because *D. nigricans* Illiger, 1802 has priority over *A. arcens* Westwood, 1847, acceptance of this association makes *D. nigricans* the valid name for this species and *A. arcens* a junior synonym.

##### Description of the queen.

This description is based on one queen associated with workers from the Van Boven collection in the RMCA (collection number Van Boven 2768). It was collected from a nest at Lamto in Ivory Coast probably on August 11, 1965 (Van Boven and Levieux 1970). ***Habitus***: Fig. 25A, overall darker and larger than *D. burmeisteri*. ***Total length***: 4.130 cm. ***Head***: Fig. 25C, HW = 5.883 mm, HL = 4.018 mm, SL = 1.916 mm, lateral sides orange, dark brown on dorsal and ventral side, surface appears slightly dull due to microreticulation, foveola evenly distributed over whole surface of head and at least as deep as wide (diameter 29 µm), no pilosity present; ***Antenna***: eleven antennomeres, funicular antennomeres dark orange and scape somewhat darker, antennal fossae pronounced, deeply impressed and round in shape, median line at the lower half V-shaped, circular impression in the upper third of median line, similar to “ocellus-like body” in *D. gribodoi* (Schöning et al. 2008b), clypeus with some setae, labrum with small median notch and some hairs, mandibles with few setae at inner side; subapical teeth missing and apical tooth slightly pointing medially, small lateral indentation like in ocular area of males, posterior angles of head drawn out backwards and ventrally. ***Mesosoma***: Fig. 25A, B, PW = 3.618 mm, PropW = 4.192 mm, HFL = 3.923 mm, colour similar to dorsal areas of head, dark brown, lighter close to sutures between segments, pronotal suture slightly impressed, ventral margin of pronotum with small hairy ridge, propodeum rectilinear, spiracle rim not visible in dorsal view; at metanotal groove with medial spine projecting backwards; surface sculpture similar to head, with microreticulation and foveolae; pleura and coxae of pro-, meso- and metathorax with yellowish pilosity at junctions, terminal segments of legs missing. ***Petiole***: Fig. 25A, B, PeW = 6.990 mm, blackish brown in colour, surface sculpture similar to head and mesosoma, no setae and without pilosity, posterior lateral lobes very conspicuous in dorsal view. ***Gaster***: Fig. 25A, B blackish brown, shinier than head and mesosoma but otherwise similar, with numerous scratches of unknown origin. ***Genitalia***: Fig. 25D, typical driver ant hypopygium similar to Gotwald (1982: 167).

##### Biology.

In our surveys, the species was found from Liberia to Ghana. The type locality is further to the West in Sierra Leone. Based on the information of habitat types at the collection localities we inferred that the species is restricted to moist forest habitats. This species is also regularly consumed by chimpanzees at Bossou, Seringbara, and Taï (Schöning et al. 2008c; Koops et al. 2015). As mentioned above, Leroux, unbeknownst to him, was dealing with two species in his long-term field study at Lamto. The colonies designated 2”0”, 2R, 52B, IB, and IE by him belong to *D. nigricans*.

### Key to the large workers of driver ants occurring in the region west of the Dahomey Gap

The key provided below is applicable to the driver ant species occurring west of the Dahomey Gap (longitude < 0.5°) and only to major workers with a pronotum width > than 1 mm. The identification is based on morphometric measurements, since there are no conspicuous qualitative characters that apply to all individuals over a large size range. The four measurements required are PW, SL, PeL, and PeW. The character descriptions are detailed in the Materials and methods section of this paper. The mathematical functions given below are derived from the analysis of data shown in Figs 9, 10. To ensure reproducibility, we recommend following suggestions made by Seifert (2002). Since driver ant colonies sometimes contain worker individuals with aberrant traits, it is necessary to take measurements of at least three individuals per nest sample and check them for consistency.

**Table:** 

1	Basisternal spines absent	**This specimen does not belong to the *Dorylus nigricans* group and cannot be identified using this key**
–	Basisternal spines present	**2 (*Dorylus nigricans* group)**
2	[SL in mm] – (0.5728 + 0.8816 • [PW in mm]) > -0.08. Antennae and legs long, head in full face view trapezoidal with lateral sides almost straight, mandibles in largest specimen very long and slender, with one subapical tooth, surface of head dull and with reticulated microsculpture, usually a general black and dull appearance.	***D. nigricans* Illiger, 1802**
–	[SL in mm] – (0.5728 + 0.8816 • [PW in mm]) < -0.08. Antennae and legs shorter, sides of the head in full face view more rounded, mandibles in largest specimen not as long and slender, with larger subapical tooth, due to less pronounced microsculpture head appears shinier, often with red areas on the body.	**3**
3	[PeW]/[PeL] > 0.75. Petiole in dorsal view trapezoid and usually with visible posterior tubercles. Head is widest close to the insertion of the mandibles, i.e. in the lower ⅓ of the head, legs shorter relative to head width, large workers (HW > 3 mm, PW > 1.4 mm) regularly present.	***Dorylus burmeisteri* Shuckard, 1840**
–	[PeW]/[PeL] < 0.75. Petiole in dorsal view narrow, with lateral sides almost parallel, head rounder, being widest halfway between hind margin and clypeus, legs longer relative to head, large workers (HW > 3 mm, PW > 1.4 mm) are rare.	***Dorylus mayri* Santschi, 1912**

## Discussion

We established the male-worker association for one of the two syntypes of *D. nigricans*, which was the first driver ant species to be described. In theory, there were two options for designating a lectotype, but only the choice we made links the name to a known worker form. The advantage is that the status of subspecific *D. nigricans* taxa based on descriptions of workers from more eastern parts of Africa can now be investigated with a clear reference.

It is curious and unfortunate that no other driver ant males were collected despite considerable sampling efforts. However, we know of three distinct driver ant male forms from the region: the *D. nigricans* lectotype and paralectotype specimens and the *D. funereus* male. Hopefully, further sampling efforts at a site where all three species are present will allow establishing the missing male-worker associations.

Our results provide the basis for more detailed ecological and behavioural studies on the three driver ant species occurring in West Africa. Based on the currently available data, *D. mayri* and *D. nigricans* appear to be rainforest specialists, while *D. burmeisteri* is more catholic in its habitat requirements. Since all three species hunt through swarm raids and are therefore expected to consume a wide variety of prey species, it will be interesting to identify the factors that allow them to coexist in humid forest areas such as Taï and Grebo.

## Supplementary Material

XML Treatment for Dorylus burmeisteri

XML Treatment for Dorylus mayri

XML Treatment for Dorylus nigricans

## Additional information

### Artificial Intelligence (AI) use

The authors accept full responsibility for the content of the manuscript, including the disclosure of any use of AI.

No AI tools were used in the preparation of this manuscript.

### Author ORCIDs

R. Weniger https://orcid.org/0009-0008-5729-2653

L. Vilhelmsen https://orcid.org/0000-0002-5593-5722

J.J. Boomsma https://orcid.org/0000-0002-3598-1609

D.J.C. Kronauer https://orcid.org/0000-0002-4103-7729

### Data availability

All of the data that support the findings of this study are available in the main text or Supplementary Information.

## Supplementary materials

Supplementary material 1 p_distances_coii Data type csv Explanation note **table SS1**. Estimates of Evolutionary Divergence between Sequences. https://binary.pensoft.net/file/1754129This dataset is made available under the Open Database License (http://opendatacommons.org/licenses/odbl/1.0/). The Open Database License (ODbL) is a license agreement intended to allow users to freely share, modify, and use this Dataset while maintaining this same freedom for others, provided that the original source and author(s) are credited. Caspar Schöning, Raphael Weniger, William Gotwald Jr., Lars Vilhelmsen, Jacobus J. Boomsma, Daniel J. C. Kronauer

Supplementary material 2 p_distance_wg_lr Data type csv Explanation note **table SS2**. Estimates of Evolutionary Divergence between Sequences. https://binary.pensoft.net/file/1754130This dataset is made available under the Open Database License (http://opendatacommons.org/licenses/odbl/1.0/). The Open Database License (ODbL) is a license agreement intended to allow users to freely share, modify, and use this Dataset while maintaining this same freedom for others, provided that the original source and author(s) are credited. Caspar Schöning, Raphael Weniger, William Gotwald Jr., Lars Vilhelmsen, Jacobus J. Boomsma, Daniel J. C. Kronauer

Supplementary material 3 morphometrics_males Data type csv https://binary.pensoft.net/file/1754131This dataset is made available under the Open Database License (http://opendatacommons.org/licenses/odbl/1.0/). The Open Database License (ODbL) is a license agreement intended to allow users to freely share, modify, and use this Dataset while maintaining this same freedom for others, provided that the original source and author(s) are credited. Caspar Schöning, Raphael Weniger, William Gotwald Jr., Lars Vilhelmsen, Jacobus J. Boomsma, Daniel J. C. Kronauer

Supplementary material 4 morphometrics_workers Data type csv https://binary.pensoft.net/file/1754132This dataset is made available under the Open Database License (http://opendatacommons.org/licenses/odbl/1.0/). The Open Database License (ODbL) is a license agreement intended to allow users to freely share, modify, and use this Dataset while maintaining this same freedom for others, provided that the original source and author(s) are credited. Caspar Schöning, Raphael Weniger, William Gotwald Jr., Lars Vilhelmsen, Jacobus J. Boomsma, Daniel J. C. Kronauer

Supplementary material 5 morphometrics_petiole_workers Data type csv https://binary.pensoft.net/file/1754133This dataset is made available under the Open Database License (http://opendatacommons.org/licenses/odbl/1.0/). The Open Database License (ODbL) is a license agreement intended to allow users to freely share, modify, and use this Dataset while maintaining this same freedom for others, provided that the original source and author(s) are credited. Caspar Schöning, Raphael Weniger, William Gotwald Jr., Lars Vilhelmsen, Jacobus J. Boomsma, Daniel J. C. Kronauer

Supplementary material 6 Sites of driver ant surveys Data type docx https://binary.pensoft.net/file/1754134This dataset is made available under the Open Database License (http://opendatacommons.org/licenses/odbl/1.0/). The Open Database License (ODbL) is a license agreement intended to allow users to freely share, modify, and use this Dataset while maintaining this same freedom for others, provided that the original source and author(s) are credited. Caspar Schöning, Raphael Weniger, William Gotwald Jr., Lars Vilhelmsen, Jacobus J. Boomsma, Daniel J. C. Kronauer

Supplementary material 7 r_scripts.r Data type r https://binary.pensoft.net/file/1754135This dataset is made available under the Open Database License (http://opendatacommons.org/licenses/odbl/1.0/). The Open Database License (ODbL) is a license agreement intended to allow users to freely share, modify, and use this Dataset while maintaining this same freedom for others, provided that the original source and author(s) are credited. Caspar Schöning, Raphael Weniger, William Gotwald Jr., Lars Vilhelmsen, Jacobus J. Boomsma, Daniel J. C. Kronauer
