# Supplementary material for: Taxonomy of African driver ants (Hymenoptera, Formicidae, Dorylus nigricans species group) west of the Dahomey Gap
Source: Zookeys. 2026 Aug 25;1290:185–226. doi: 10.3897/zookeys.1290.192534 (PMC13535510; doi:10.3897/zookeys.1290.192534)
Supplement: Supplementary file 1 [file zookeys-1290-185_article-192534__-s006.docx]

Supplementary Table 1: List of study sites of driver ant surveys, their habitat types and the occurring driver ant species

| **Country** | **Site** | **Main habitat type (according to A.P.E.S. Wiki)** | **Number of samples collected** | **Name of collectors** | ***Dorylus nigricans* present?** | ***Dorylus burmeisteri* present?** | ***Dorylus mayri* present?** |
| --- | --- | --- | --- | --- | --- | --- | --- |
| Guinea-Bissau | Boe | savanna and gallery forests | 46 | Els Ton, Joost van Schijndel |  | X |  |
| Guinea | Bakoun | savanna, shrubland, subtropical/tropical dry forest | 12 | Anthony Agbor |  | X |  |
|  | Sangaredi | subtropical/tropical heavily degraded former forest | 10 | Adama Keita, Conde Kabinet, Beavogui Assane Sefou |  | X |  |
|  | Seringbara | primary and secondary rain forest, montane grasslands | several hundreds | Kathelijne Koops | X | X | X |
|  | Sobeya | savanna, shrubland, subtropical/tropical dry forest | 64 | Beavogui Assane Sefou, Gregory Brazzola |  | X |  |
|  | Sobory | savanna, shrubland, subtropical/tropical dry forest | 28 | Abdoulaye Toure, Anthony Agbor |  | X |  |
| Senegal | Dindefelo | savanna, shrubland, subtropical/tropical dry forest | 21 | Amanda Barciela, Anais Aviles, Gimena Coppola, Juan Manueal Garcia, Manuel Llana, Paula Álvarez, Veronica Moreno, |  | X |  |
|  | Fongoli |  | 3 | Jill Pruetz, Stephanie Bogart, William McGrew |  | X |  |
| Sierra Leone | Outamba Kilimi National Park | subtropical/tropical dry forest, subtropical/tropical moist lowland forest | 27 | Bradley Larson |  | X |  |
| Liberia | East Nimba | subtropical/tropical moist montane forest | 14 | Sergio Marrocoli, Sorrel Jones | X | X | X |
|  | Grebo National Park | subtropical/tropical moist lowland forest | 54 | Sergio Marrocoli | X | X | X |
|  | 47 sites (nationwide survey, Twehe et al. 2014) | various | 37 | - | X | X | X |
| Ivory Coast | Comoé National Park | savanna, subtropical/tropical dry forest | 26 | Christian Kost, Jan Beck, Juan Lapuente, Paco Kouamé |  | X |  |
|  | GEPRENAF | savanna, subtropical/tropical dry forest | 20 | Alex Tickle |  | X |  |
|  | Djouroutou | subtropical/tropical moist lowland forest (adjacent to Taï National Park) | 6 | Juan Lapuente | X |  |  |
|  | Lamto | forest-savanna-mosaic | 4 | Mouhamadou Koné, Souleymane Konate |  | X |  |
|  | Mont Sângbé | Savanna woodland with some gallery forests | 22 | Heather Cohen, Jodie Preece |  | X |  |
|  | Taï National Park | subtropical/tropical moist lowland forest | > 100 | Amelia Meier, Caspar Schöning, Juan Lapuente, Lydia Luncz, Tobias Deschner, Yasmin Möbius | X | X | X |
| Ghana | Bobiri | moist tropical forest | 19 | Lois Fisch, Loyd Davis, Mark Moffett, William Gotwald Jr | X | X |  |
|  | Efutu near Cape Coast | coastal savanna and forest fragments | 2 | Lois Fisch, William Gotwald Jr |  | X |  |
|  | Kakum National Park | moist evergreen forest and seasonal dry semi-deciduous forest | 1 | Lois Fisch, William Gotwald Jr |  |  | X |
|  | New Tafo | moist forest, cocoa plantations | 3 | Lois Fisch, William Gotwald Jr | X | X |  |

References:

A.P.E.S. (2025) A.P.E.S. Wiki. <https://wiki.iucnapesportal.org/index.php/Western_chimpanzee>

Tweh CG, Lormie MM, Kouakou CY, Hillers A, Kühl HS, Junker J (2014) Conservation status of chimpanzees *Pan troglodytes verus* and other large mammals in Liberia: a nationwide survey. Oryx 49:710-718. <https://doi.org/10.1017/S0030605313001191>
